# The interaction of secreted phospholipase A_2_-IIA with the microbiota alters its lipidome and promotes inflammation

**DOI:** 10.1172/jci.insight.152638

**Published:** 2022-01-25

**Authors:** Etienne Doré, Charles Joly-Beauparlant, Satoshi Morozumi, Alban Mathieu, Tania Lévesque, Isabelle Allaeys, Anne-Claire Duchez, Nathalie Cloutier, Mickaël Leclercq, Antoine Bodein, Christine Payré, Cyril Martin, Agnes Petit-Paitel, Michael H. Gelb, Manu Rangachari, Makoto Murakami, Laetitia Davidovic, Nicolas Flamand, Makoto Arita, Gérard Lambeau, Arnaud Droit, Eric Boilard

**Affiliations:** 1 CHU de Québec-Université Laval Research Center, Department of Microbiology, Infectiology and Immunology, Quebec City, Quebec, Canada.; 2 ARThrite Research Center, University Laval, Quebec City, Quebec, Canada.; 3 CHU de Québec-Université Laval Research Center, Endocrinology and Nephrology Axis, Quebec City, Quebec, Canada.; 4 Laboratory for Metabolomics, RIKEN Center for Integrative Medical Sciences, Yokohama, Japan.; 5 Division of Physiological Chemistry and Metabolism, Graduate School of Pharmaceutical Sciences, Keio University, Tokyo, Japan.; 6 Côte d’Azur University, The French National Centre for Scientific Research, Institute of Molecular and Cellular Pharmacology, UMR7275, Valbonne Sophia Antipolis, France.; 7 The Research Center of the University Institute of Cardiology and Pneumology of Quebec, Quebec City, Quebec, Canada.; 8 Department of Chemistry, University of Washington, Seattle, Washington, USA.; 9 CHU de Québec-Université Laval Research Center, Neurosciences Axis, Quebec City, Quebec, Canada.; 10 Laboratory of Microenvironmental and Metabolic Health Science, Center for Disease Biology and Integrative Medicine, Graduate School of Medicine, The University of Tokyo, Tokyo, Japan.; 11 Cellular and Molecular Epigenetics Laboratory, Graduate School of Medical Life Science, Yokohama-City University, Yokohama, Japan.

**Keywords:** Inflammation, Microbiology, Arthritis, Molecular pathology, Mouse models

## Abstract

Secreted phospholipase A_2_-IIA (sPLA_2_-IIA) hydrolyzes phospholipids to liberate lysophospholipids and fatty acids. Given its poor activity toward eukaryotic cell membranes, its role in the generation of proinflammatory lipid mediators is unclear. Conversely, sPLA_2_-IIA efficiently hydrolyzes bacterial membranes. Here, we show that sPLA_2_-IIA affects the immune system by acting on the intestinal microbial flora. Using mice overexpressing transgene-driven human sPLA_2_-IIA, we found that the intestinal microbiota was critical for both induction of an immune phenotype and promotion of inflammatory arthritis. The expression of sPLA_2_-IIA led to alterations of the intestinal microbiota composition, but housing in a more stringent pathogen-free facility revealed that its expression could affect the immune system in the absence of changes to the composition of this flora. In contrast, untargeted lipidomic analysis focusing on bacteria-derived lipid mediators revealed that sPLA_2_-IIA could profoundly alter the fecal lipidome. The data suggest that a singular protein, sPLA_2_-IIA, produces systemic effects on the immune system through its activity on the microbiota and its lipidome.

## Graphical abstract

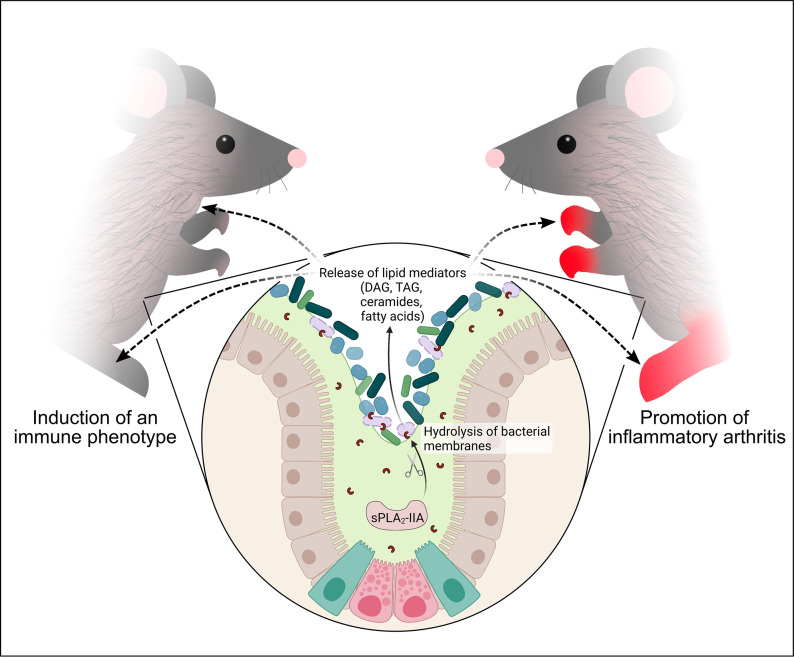

## Introduction

The mammalian digestive tract harbors trillions of microorganisms, collectively known as the microbiota (1). Cohabitation of the commensal microbiota with cells that populate the intestinal epithelium plays a central role in host metabolism and acts as an important barrier that prevents the implantation of pathogens (2–4). Moreover, the intestinal microbiota plays key roles for the development and homeostasis of the immune system and affects both innate and adaptive immunity (5, 6).

Dysbiosis is associated with alterations in microbial composition and functional changes in the microbiota. Dysbiosis notably contributes to the advent or exacerbation of inflammation, such as in inflammatory bowel diseases (7, 8), but it can also have systemic consequences that expand beyond the intestinal tract. As such, dysbiosis has been associated with a wide variety of diseases, including autoimmune, metabolic, and neurological disorders (9, 10). However, it is still unclear whether dysbiosis is a cause or a consequence of these diseases (11). Therefore, it is critical to identify the factors that promote dysbiosis and to define their contribution to inflammatory processes.

Exogenous factors such as diet, alcohol abuse, antibiotics and other medications, and invading pathogenic microorganisms can impact microbiota composition (12–16). Moreover, genetic factors and endogenous antimicrobial proteins play prominent roles in shaping and maintaining the microbiota composition (17, 18). Antimicrobial peptides (AMPs) are components of the innate immune system with antimicrobial activities (19). They are typically present on mucosal surfaces, and their expression may increase in response to infection (20). In vertebrates, defensins, cathelicidins, the S100 family, the RNase A superfamily, regenerating islet-derived III (RegIII) C-type lectins, and peptidoglycan-recognition proteins are some of the best described AMPs and are suggested to impact the balance of the host microbiota (19, 21).

Secreted phospholipase A_2_-IIA (sPLA_2_-IIA) also belongs to the family of AMPs but has not been as extensively studied. This is, in part, because this enzyme is absent in C57BL/6 and other commonly used mouse models due to a natural frameshift mutation in the *Pla2g2a* gene (22). sPLA_2_-IIA is a 14 kDa protein originally identified in the synovial fluid of patients with rheumatoid arthritis (RA), platelets, and intestine that is induced in both infectious and noninfectious inflammatory conditions (23–33). It hydrolyzes membrane phospholipids to release free fatty acids and lysophospholipids, and it is thought to contribute to the generation of potent lipid mediators like eicosanoids generated from arachidonic acid (25, 34). Though C57BL/6 mice lack sPLA_2_-IIA, transgenic expression of human sPLA_2_-IIA in C57BL/6 mice (sPLA_2_-IIA^TGN^) revealed that this enzyme enhances arthritis severity (35). Intriguingly, lipids comprising the plasma membrane of eukaryotic cells are poor substrates of sPLA_2_-IIA (36, 37), challenging the concept that this enzyme could contribute to sterile inflammation. The enzyme shows high selectivity toward phosphatidylethanolamine and anionic phospholipids such as phosphatidylglycerol over phosphatidylcholine (36–39). While these phospholipids are minor constituents of the plasma membrane outer leaflet, they are accessible to the enzyme on Gram-positive bacterial membranes, and as such, the bactericidal activity of sPLA_2_-IIA toward these bacteria has been well established (40–43).

Under physiological conditions in humans, sPLA_2_-IIA is mainly expressed by specialized secretory epithelial cells such as those from the posterior lobe and periurethral glands of the prostate and lacrimal gland cells (44–46). Moreover, while it is unknown whether expression of sPLA_2_-IIA may affect the expression of other AMPs, sPLA_2_-IIA is also liberated alongside these peptides by Paneth cells in the intestine (21, 47). Despite its low expression relative to other AMPs (48), sPLA_2_-IIA is more potent at hydrolyzing Gram-positive bacterial membrane, requiring only nanomolar concentrations to efficiently eliminate certain bacteria (43, 49). Under infectious and inflammatory conditions, sPLA_2_-IIA expression is highly induced in numerous cell types, including endothelial, hepatic, renal, smooth muscle, macrophages, mast cells, platelets, astrocytes, and glial cells (34, 45, 50–63). The serum concentration of sPLA_2_-IIA can increase up to 1000-fold during sepsis, to reach concentrations of ~5 μg/mL (~350 nM) (64), and recent studies revealed that it is also induced in COVID-19 patients (65). Investigation of the gene promoter revealed that sPLA_2_-IIA is induced by proinflammatory cytokines such as IL-1β, IL-6, and TNF-α (66–69), while activation of TLR and nucleotide-binding oligomerization domain-like receptors (NLR) by pathogen-associated molecular patterns (PAMPs) also trigger sPLA_2_-IIA expression through the NF-κB pathway (70–74). Thus, stimuli relevant to both sterile inflammation and infection promote overexpression of sPLA_2_-IIA.

Multiple lines of evidence point to a crosstalk between sPLA_2_-IIA and the intestinal microbiome. Depletion of the microbiota in BALB/c mice naturally expressing sPLA_2_-IIA reduces its expression by Paneth cells (75). Moreover, the colonization of germ-free C3H mice with the microbiota of conventionally housed mice increases the levels of sPLA_2_-IIA RNA transcripts in the intestine (76), suggesting that the microbiota modulates sPLA_2_-IIA intestinal expression. In patients with cystic fibrosis, the pathogenic bacteria *Pseudomonas aeruginosa* was shown to eliminate and outgrow *Staphylococcus aureus* through the induction of sPLA_2_-IIA expression in lungs (77). Colonization with *P*. *aeruginosa* in patients is associated with poor prognosis (78, 79), which is concordant with the accelerated mortality of infected sPLA_2_-IIA^TGN^ mice relative to control mice (77). Furthermore, the oral pathobiont *Porphyromonas gingivalis* induces sPLA_2_-IIA, which may contribute to oral dysbiosis during periodontal disease (80). In summary, sPLA_2_-IIA is induced by bacteria and may contribute to the enhancement of inflammatory processes during infection. While sPLA_2_-IIA is also induced in numerous inflammatory conditions in the absence of infection, it remains to be investigated whether sPLA_2_-IIA affects the microbiota, thereby contributing to sterile inflammation. In this study, we evaluated the impact of the sPLA_2_-IIA–microbiota interplay on the homeostasis of the immune system and the promotion of autoimmune inflammatory arthritis.

## Results

### Transgenic overexpression of sPLA_2_-IIA disrupts immune homeostasis.

**Figure 1:**
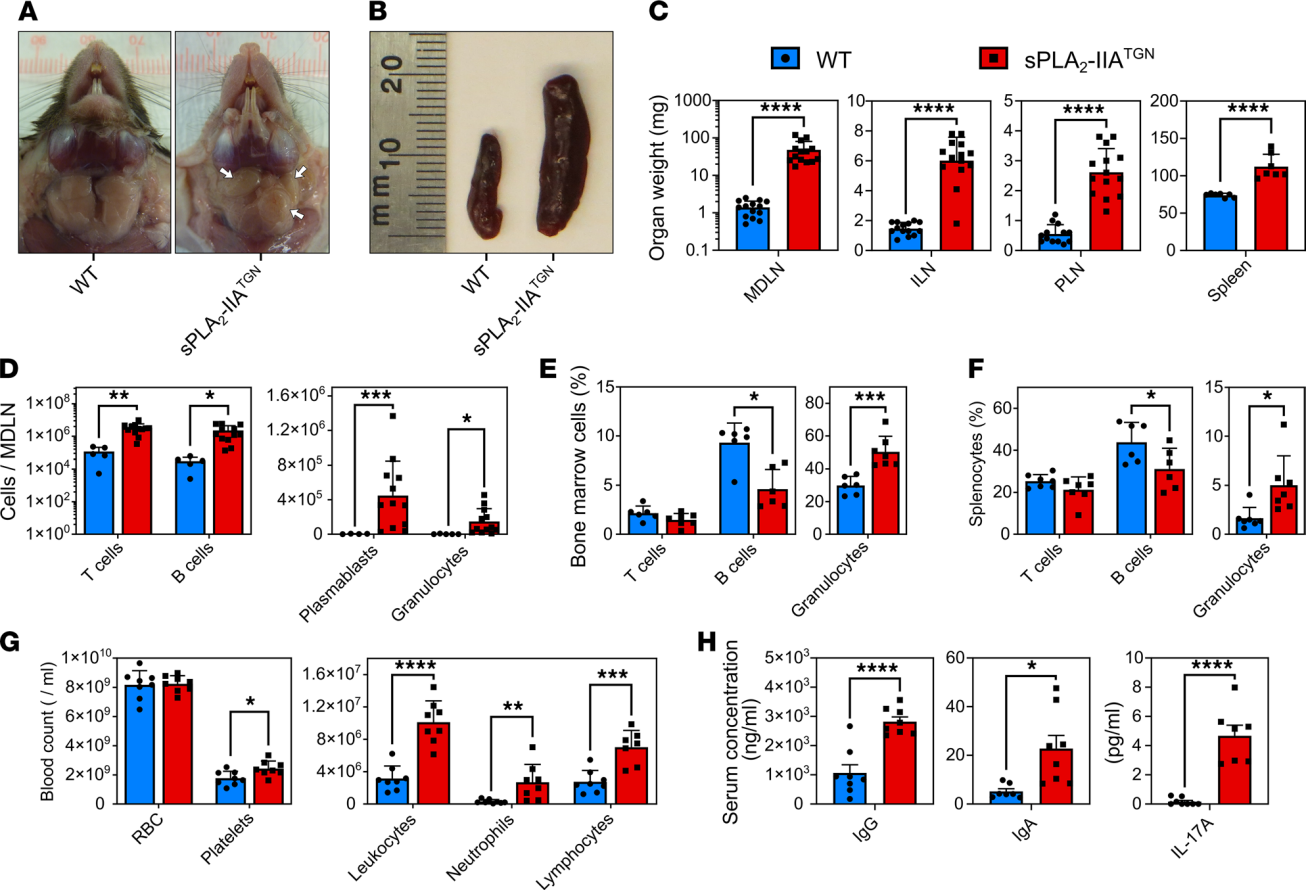
Spontaneous induction of immune disturbances in sPLA_2_-IIA^TGN^ mice. (**A** and **B**) Representative mandibular lymph nodes (MDLN, white arrows) and spleen of 8-month-old WT and sPLA_2_-IIA^TGN^ mice housed in a SPF animal facility (*n* = 8). (**C**) Weight of MDLNs (*n* = 14), inguinal lymph nodes (ILN, *n* = 14), popliteal lymph nodes (PLN, *n* = 13–14), and the spleen (*n* = 7) from both mouse groups. (**D**–**F**) Flow cytometric analysis with markers targeting T cells (CD3^+^B220^–^), B cells (B220^+^CD3^–^), plasmablasts (CD19^+^CD138^+^), and granulocytes (Gr1^+^). (**D**–**F**) Cell counts are represented for MDLNs (*n* = 5 WT and 12 sPLA_2_-IIA^TGN^), and cell proportions are represented for the BM (*n* = 6–7) and spleen (*n* = 6–7) of 8-month-old WT and sPLA_2_-IIA^TGN^ mice. (**G**) Blood composition of both mouse groups determined by complete blood count (*n* = 8). (**H**) Quantification of type G (IgG) and type A (IgA) immunoglobulin by ELISA (*n* = 8) and IL-17A by cytometric bead array (*n* = 7–8) in the serum of WT and sPLA_2_-IIA^TGN^ mice. Data from 7 separate experiments are presented as mean ± SEM. Statistical analysis involved unpaired t test. *P < 0.05, **P < 0.01, ***P < 0.001, ****P < 0.0001.

The study was initiated by the serendipitous observation of spontaneous swelling in the neck of sPLA_2_-IIA^TGN^ mice housed in a specific pathogen–free (SPF) facility for an extended duration, by approximately 8 months of age. Upon palpating the swollen area, marked large and solid lumps were detected. This was never observed in C57BL/6J WT mice, which lack sPLA_2_-IIA due to a natural frameshift mutation (22). Invasive investigation led us to the observation that the palpable lumps reflected the presence of enlarged mandibular lymph nodes (MDLN) in the transgenic animals (Figure 1A). Further inspection also revealed enlargement of the spleen and multiple other lymph nodes including inguinal lymph nodes (ILN) and popliteal lymph nodes (PLN) (Figure 1, B and C), suggestive of changes in immune populations in lymphoid organs of these mice.

To further investigate this perturbation, flow cytometry was used to determine the numbers and frequencies of various immune cell types. In MDLNs, we found a marked increase in the absolute counts of T cells, B cells, plasmablasts, and granulocytes (Figure 1D). There was only an increase in the frequency of plasmablasts in lymph nodes of sPLA_2_-IIA^TGN^ mice, while frequencies of other cell types were not impacted (Supplemental Figure 1A; supplemental material available online with this article; https://doi.org/10.1172/jci.insight.152638DS1). Analysis of BM and spleen revealed a significant decrease in the frequency of B cells and a tendency for reduced T cell frequency (Figure 1, E and F). Conversely, granulocyte proportions were increased in the BM and spleen of transgenic animals (Figure 1, E and F, and Supplemental Figure 1B). Altogether, these data indicate a systemic immune process affecting multiple immune cell compartments upon transgenic expression of sPLA_2_-IIA.

Analysis of circulating blood in 8-month-old sPLA_2_-IIA^TGN^ mice revealed a modest, but significant, increase in platelets, as well as a 3-fold increase in leukocyte counts — particularly neutrophils and lymphocytes (Figure 1G). Moreover, higher levels of circulating IgG and IgA were observed in sPLA_2_-IIA^TGN^ mice (Figure 1H), and this is consistent with the accumulation of plasmablasts in MDLNs. Intriguingly, among 6 different cytokines measured in blood (IL-1β, IL-2, IL-6, IL-10, IL-17A, and TNF-α) (Supplemental Figure 1C), only IL-17A was found to be increased in sPLA_2_-IIA^TGN^ mice (Figure 1H), perhaps suggesting that Th17 responses were exacerbated in these animals.

### The immune phenotype is independent of cytosolic phospholipase A_2_-α and 12-lipoxygenase.

It was previously shown that sPLA_2_-IIA contributes to inflammation via the activation of cytosolic phospholipase A_2_-α (cPLA_2_) and subsequent production of inflammatory eicosanoids (33, 81–84). Results from in vivo and in vitro studies have also determined that, once it is released by sPLA_2_-IIA from platelet-derived extracellular vesicles, arachidonic acid is readily metabolized into 12-hydroxyeicosatetraenoic acid through the activity of 12-lipoxygenase (12-LO) conveyed in platelet extracellular vesicles (85). We thus examined the phenotypes of cPLA_2_^−/−^ and *Alox12*^−/−^ (lacking 12-LO) mice crossed with sPLA_2_-IIA^TGN^ mice. We found that the immune disturbances identified in sPLA_2_-IIA^TGN^ mice persisted in sPLA_2_-IIA^TGN^:cPLA_2_^−/−^ and sPLA_2_-IIA^TGN^:*Alox12*^−/−^ mice (Supplemental Figure 2), thus ruling out the involvement of cPLA_2_ and 12-LO.

### sPLA_2_-IIA alters the microbiota composition.

**Figure 2:**
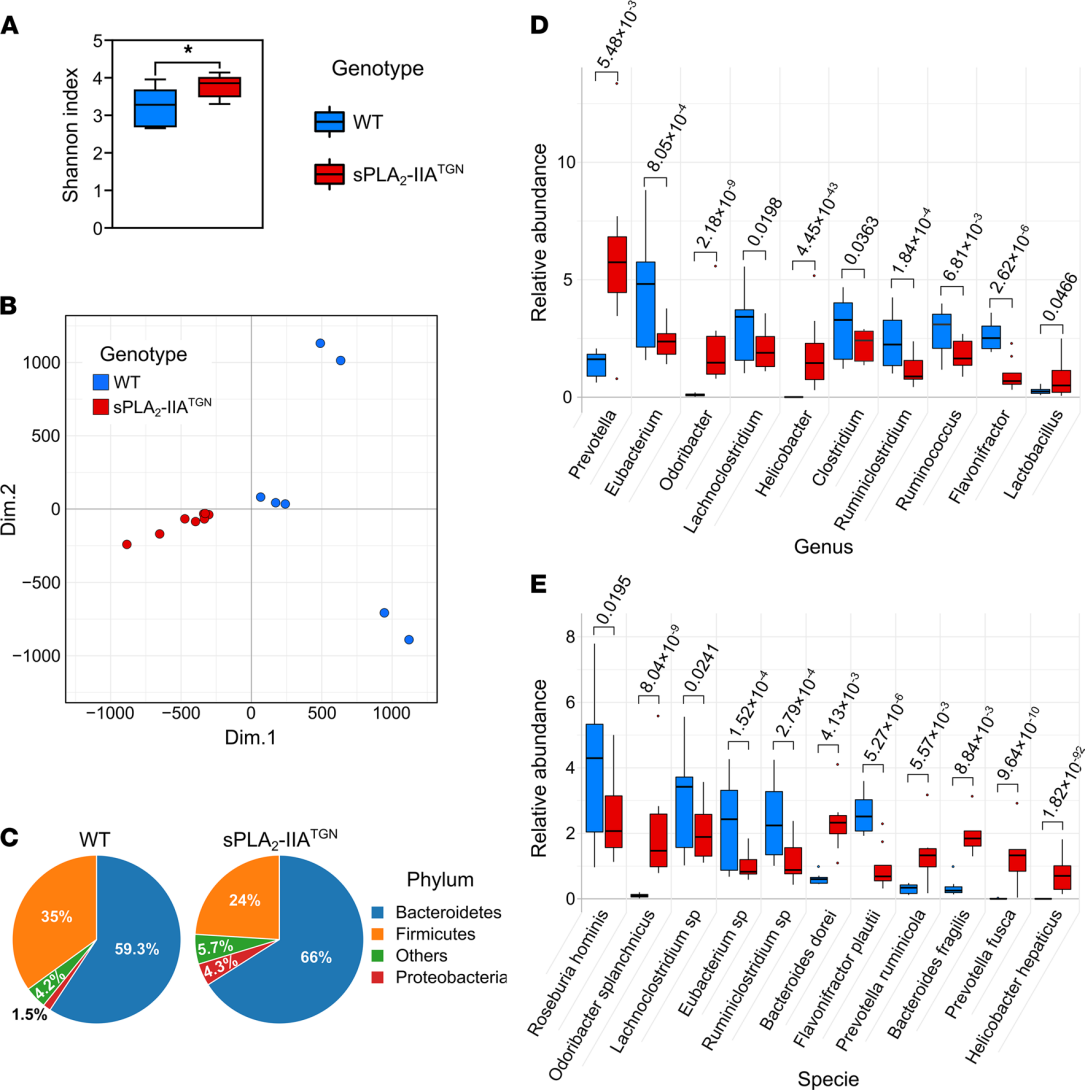
sPLA_2_-IIA^TGN^ mice housed in a SPF facility present an altered intestinal flora. A whole-genome shotgun sequencing approach was used to identify the bacterial composition of fecal samples from 8-month-old WT and sPLA_2_-IIA^TGN^ mice housed in a SPF animal facility. (**A**) α-Diversity (Shannon index) of the fecal microbiomes in each group (*n* = 7–8). (**B**) Principal component analysis comparing the composition of these microbiomes. (**C**) Representation of the relative abundance of the most abundant phyla in each group. (**D** and **E**) Most abundant and differentially enriched genera and species in WT and sPLA_2_-IIA^TGN^ mice based on a differential enrichment analysis. (**A**, **D**, and **E**) Data are presented as boxes representing the median and quartiles, with whiskers extending up to 1.5 interquartile range. Statistical analysis included the following: (**A**) unpaired *t* test and (**D** and **E**) Wald test with *P* value corrected by Benjamini-Hochberg FDR procedure. In **E**, when analysis could not identify the species, “sp” was added to the identified genus. *P < 0.05.

Another possible mechanism by which sPLA_2_-IIA could promote inflammation would be through the alteration of the microbiota composition via its antimicrobial activity toward this flora. Therefore, using DNA extracted from sPLA_2_-IIA^TGN^ and WT feces, we performed a whole-genome shotgun sequencing and compared the flora from both groups of mice. In addition to the slightly higher α-diversity of species in sPLA_2_-IIA^TGN^ mice (Figure 2A), the distribution of the microbiomes of all tested mice using a principal component analysis (PCA) distinguished the sPLA_2_-IIA^TGN^ and WT microbiomes, pointing to a modulated microbiota due to sPLA_2_-IIA expression (Figure 2B). This was confirmed using a differential enrichment analysis where notable alterations at the phylum, genus, and species levels were observed (Figure 2, C–E). Among the genera displaying the highest discrepancies between the groups, *Prevotella*, *Odoribacter*, and *Helicobacter* were found at elevated abundances in sPLA_2_-IIA^TGN^ mice, while *Eubacterium*, *Lachnoclostridium*, and *Clostridium* were more abundant when sPLA_2_-IIA was not expressed.

### Housing conditions impact sPLA_2_-IIA–mediated immune phenotype.

The mice were initially housed in a SPF animal facility that fulfilled the standard category for research, but they were rederived in a new animal facility with higher standards (Elite SPF+, hereafter referred to as Elite) at our institution. Some of the key differences between the facilities include: more restricted access to the facility, increased personal protective equipment standards, mandatory shower before entering, obligation to wear sterilized clothing, additional animal chow sterilization steps, and a more stringent pathogen exclusion list (Supplemental Table 1). To assess the development of the spontaneous immune phenotype, sPLA_2_-IIA^TGN^ and WT mice housed under these conditions were examined biweekly for the occurrence of swelling and the presence of lumps. Subtle swelling in the neck of sPLA_2_-IIA^TGN^ mice was noticed at around 10–12 weeks of age and was accompanied by small lumps at around 28 weeks. The swelling and lumps became readily noticeable when the mice reached approximately 40 weeks of age. The phenotype also appeared to somewhat stabilize at this time point, with a very slight increase in severity as mice continued to age (Supplemental Figure 3A).

**Figure 3:**
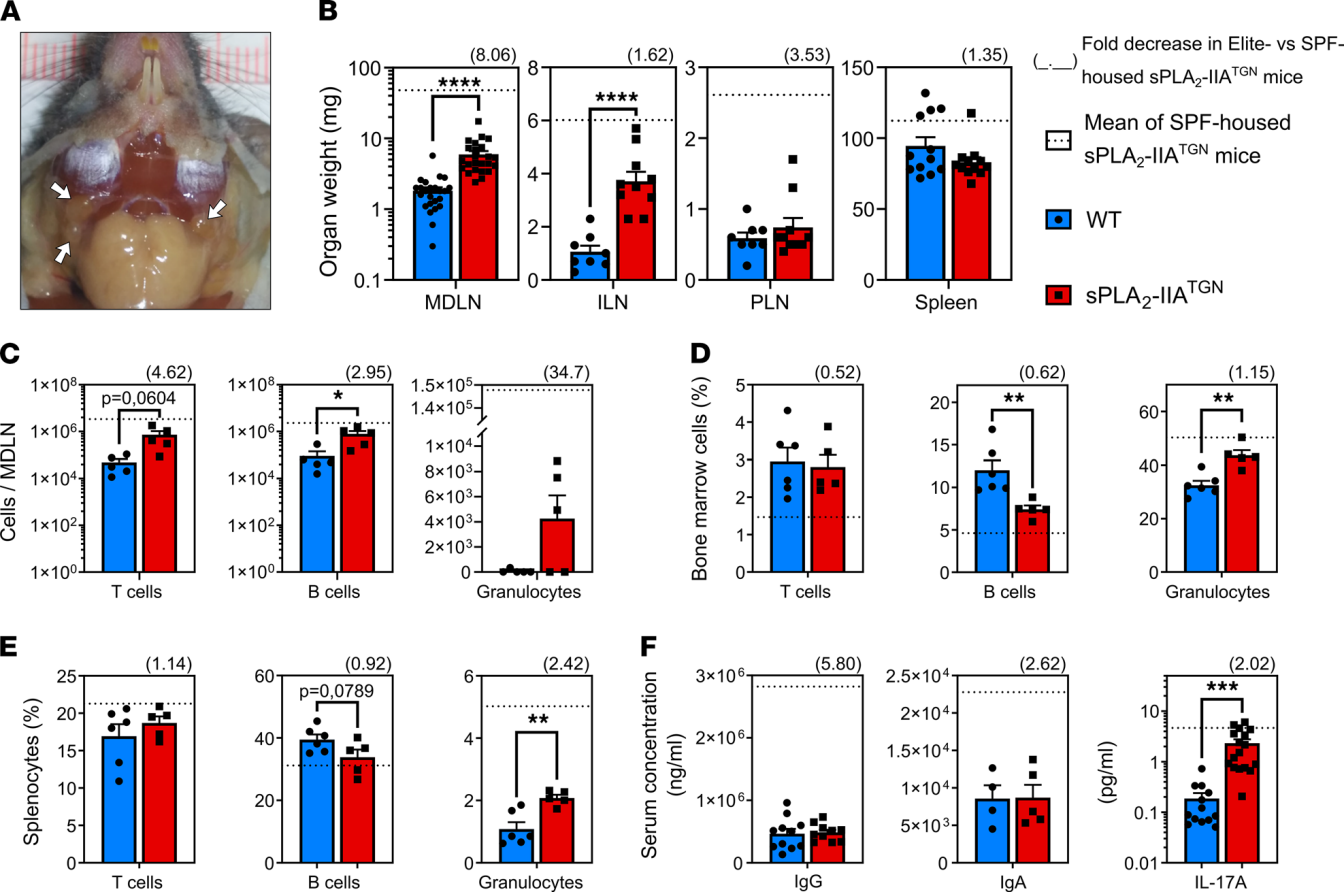
Impact of the housing environment on the immune phenotype. WT and sPLA_2_-IIA^TGN^ mice were housed in an Elite SPF+ animal facility for 14 months before the severity of the immune phenotype was assessed. (**A**) Representative mandibular lymph nodes (MDLN, white arrows) of sPLA_2_-IIA^TGN^ mice (*n* = 12). (**B**) Weight of MDLNs (*n* = 24), ILNs (*n* = 8–10), PLNs (*n* = 8–10), and spleen (*n* = 12) of both mouse groups. (**C**–**E**) Flow cytometric analysis with markers targeting T cells (CD3^+^CD19^–^), B cells (CD19^+^CD3^–^), and granulocytes (Gr1^+^). (**C**–**E**) Counts are displayed for MDLNs (*n* = 5), and the proportion of each cell type is displayed for the BM (*n* = 5–6) and spleen (*n* = 5–6) of WT and sPLA_2_-IIA^TGN^ mice. (**F**) Quantification of IgG (*n* = 10–11) and IgA (*n* = 5) by ELISA and IL-17A (*n* = 13–17) by cytometric bead array in the serum of WT and sPLA_2_-IIA^TGN^ mice. (**B**–**E**) Fold decrease of sPLA_2_-IIA^TGN^ mice housed in the Elite environment compared with sPLA_2_-IIA^TGN^ mice housed in the SPF animal facility is represented as a number in parentheses over each graph. Dotted line represents mean of sPLA_2_-IIA^TGN^ mice housed in the SPF animal facility (see Figure 1). Data from 3–4 separate experiments are presented as mean ± SEM. Statistical analysis included unpaired *t* test. *P < 0.05, **P < 0.01, ***P < 0.001, ****P < 0.0001.

As the phenotype generally appeared less pronounced in the Elite environment, mice were housed for up to 60 weeks before immune phenotyping was performed, similarly to mice housed in the SPF facility. Although MDLNs and ILNs from sPLA_2_-IIA^TGN^ mice housed in the Elite facility were still enlarged in comparison with WT mice, their size was reduced compared with that of mice housed in the SPF facility (Figure 3, A and B). Moreover, this enlargement was completely abolished in the PLNs and spleens (Figure 3B). Concordant with the reduced weight of MDLNs, the increase in T cell and B cell counts in these organs was also diminished compared with that in SPF mice (Figure 3C and Supplemental Figure 3B). An elevated proportion of granulocytes in the BM and spleen of transgenic animals was still observed in comparison with WT animals, but this level also appeared reduced in comparison with what was observed in the SPF environment (Figure 3, D and E, and Supplemental Figure 3C). No differences in circulating IgG and IgA were observed between WT and transgenic animals in the Elite environment (Figure 3F). Circulating cytokines were quantified to gain better insight into the T cell response in sPLA_2_-IIA^TGN^ mice. With the exception of IL-17A, whose increase in the presence of sPLA_2_-IIA almost reached statistical significance (*P* = 0.0873) (Supplemental Figure 4) and was confirmed in a larger set of animals (Figure 3F), no modulation was measured in the concentration of the other 17 quantified cytokines, namely GM-CSF, IFN-γ, IL-1α, IL-1β, IL-2, IL-4, IL-5, IL-6, IL-7, IL-10, IL-12p70, IL-13, CXCL1, CXCL5, MCP-1, MIP-2, and TNF-α. However, sPLA_2_-IIA^TGN^:IL-17A^−/−^ mice housed for more than 6 months under Elite conditions still presented the identified immune phenotype (Supplemental Figure 5), thus indicating that IL-17A is dispensable in its development.

Multiple bacteria have been reported to regulate sPLA_2_-IIA expression (43, 75, 77, 80). Thus, we investigated whether the concentration of sPLA_2_-IIA was modulated between the different housing conditions in this study. Despite the significant reduction in the severity of the immune phenotype in mice housed in the Elite facility, the concentrations and activity of circulating sPLA_2_-IIA were similar in both environments (Supplemental Figure 3, D and E). Moreover, while the immune phenotype was only apparent in middle-aged mice, the serum levels of sPLA_2_-IIA remained unchanged in mice between 5 and 14 months of age (Supplemental Figure 3D). Thus, multiple aspects that characterized the sPLA_2_-IIA–mediated stimulation of the immune phenotype appeared to be highly dependent upon environmental factors. As our SPF animal facility became unavailable at that time, mice housed under Elite conditions were utilized for all subsequent experiments unless stated otherwise.

### The microbiota is implicated in the sPLA_2_-IIA–mediated immune phenotype.

**Figure 4:**
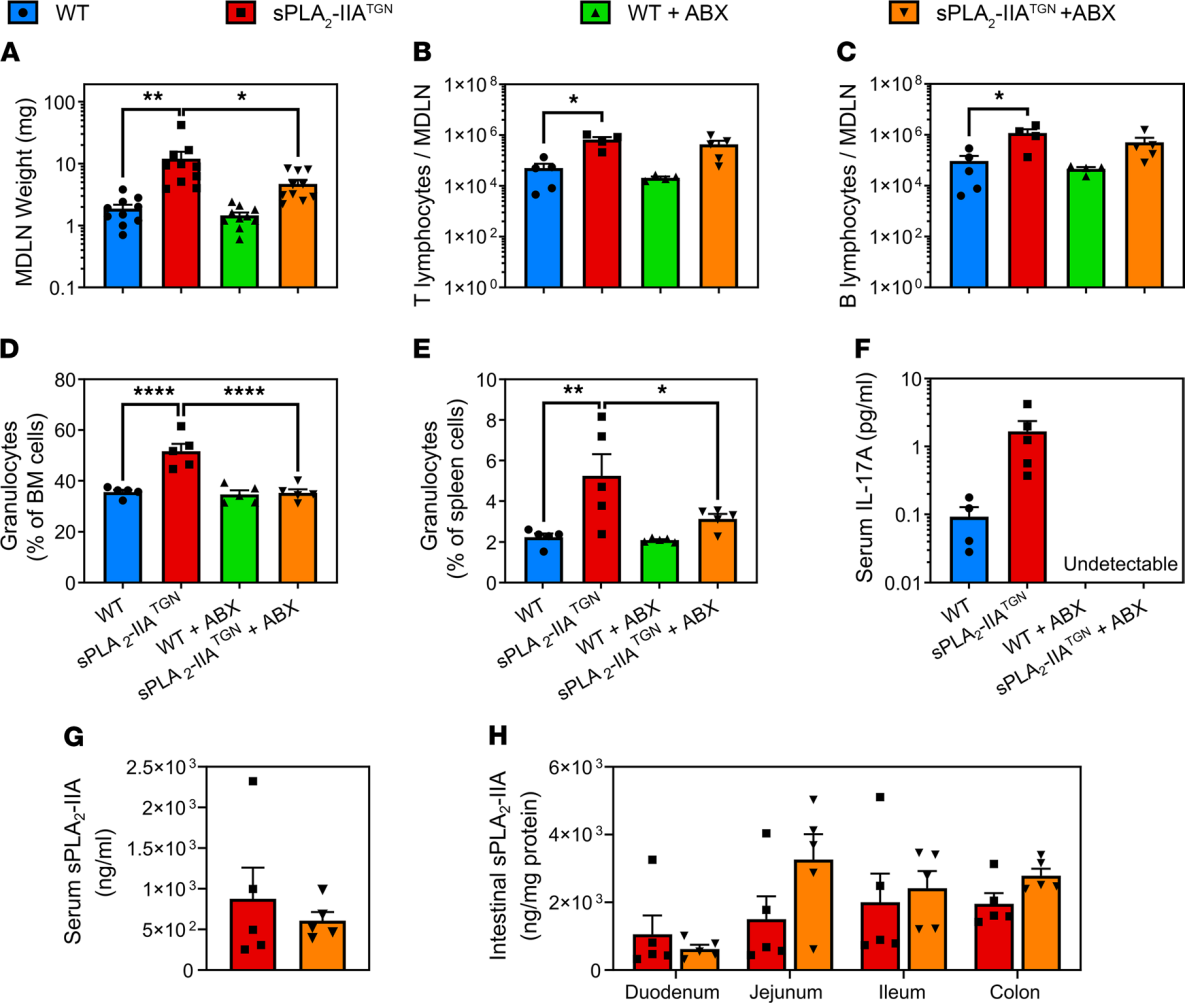
The depletion of the intestinal flora reduces the severity of the immune phenotype. The microbiota of 1-year-old WT and sPLA_2_-IIA^TGN^ mice housed in an Elite SPF+ animal facility was depleted using broad-spectrum antibiotics for 6 weeks prior to assessment of the immune phenotype. (**A**) Weight of MDLNs following antibiotic treatment (*n* = 10). (**B**–**E**) Flow cytometric analysis with markers targeting T cells (CD3^+^CD19^–^), B cells (CD19^+^CD3^–^), and granulocytes (Gr1^+^) (*n* = 5). T lymphocyte and B lymphocyte counts in MDLNs are shown, and the proportion of granulocytes in the BM and spleen of WT and sPLA_2_-IIA^TGN^ mice treated or not with antibiotics is displayed. (**F**) Dosage of IL-17A by cytometric bead array in the serum of all mouse groups (*n* = 5). (**G** and **H**) Concentration of sPLA_2_-IIA quantified by time-resolved fluoroimmunoassay in serum and intestinal lysates of sPLA_2_-IIA^TGN^ mice treated or not with antibiotics (*n* = 5). Data from 1 experiment are presented as mean ± SEM. Statistical analysis included 1-way ANOVA with Dunnett’s multiple comparisons test. *P < 0.05, **P < 0.01, ****P < 0.0001.

We assessed the actual contribution of the intestinal flora to the development of the immune process. One-year-old sPLA_2_-IIA^TGN^ and WT mice were treated with broad-spectrum antibiotics (ABX) by oral gavage (75) for 6 weeks, to achieve near complete depletion of intestinal flora (>99.9%) as shown by quantification of 16S rRNA genes in fecal samples (Supplemental Figure 6). Microbiota depletion resulted in a significant reduction in the size of MDLNs in sPLA_2_-IIA^TGN^ mice (Figure 4A). While there was only a tendency for reduction of T cell and B cell counts in MDLNs from ABX-treated sPLA_2_-IIA^TGN^ mice (Figure 4, B and C), the lymphocytes counts in ABX-treated mice were not significantly increased in sPLA_2_-IIA^TGN^ mice compared with WT mice. The treatment also completely abolished the increase in granulocytes in the spleen and BM of sPLA_2_-IIA^TGN^ mice (Figure 4, D and E). Moreover, although circulating IL-17A was reduced to undetectable levels in both ABX-treated WT and sPLA_2_-IIA^TGN^ mice (Figure 4F), concentration and activity of sPLA_2_-IIA both in the circulation and intestine remained unaltered by ABX treatment (Figure 4, G and H, and Supplemental Figure 7, A and B). Together, the data suggest that the microbial flora may play an active part in the sPLA_2_-IIA–mediated immune phenotype and that this is at least partially reversible.

### Role of the sPLA_2_-IIA–microbiota interplay in susceptibility to arthritis.

**Figure 5:**
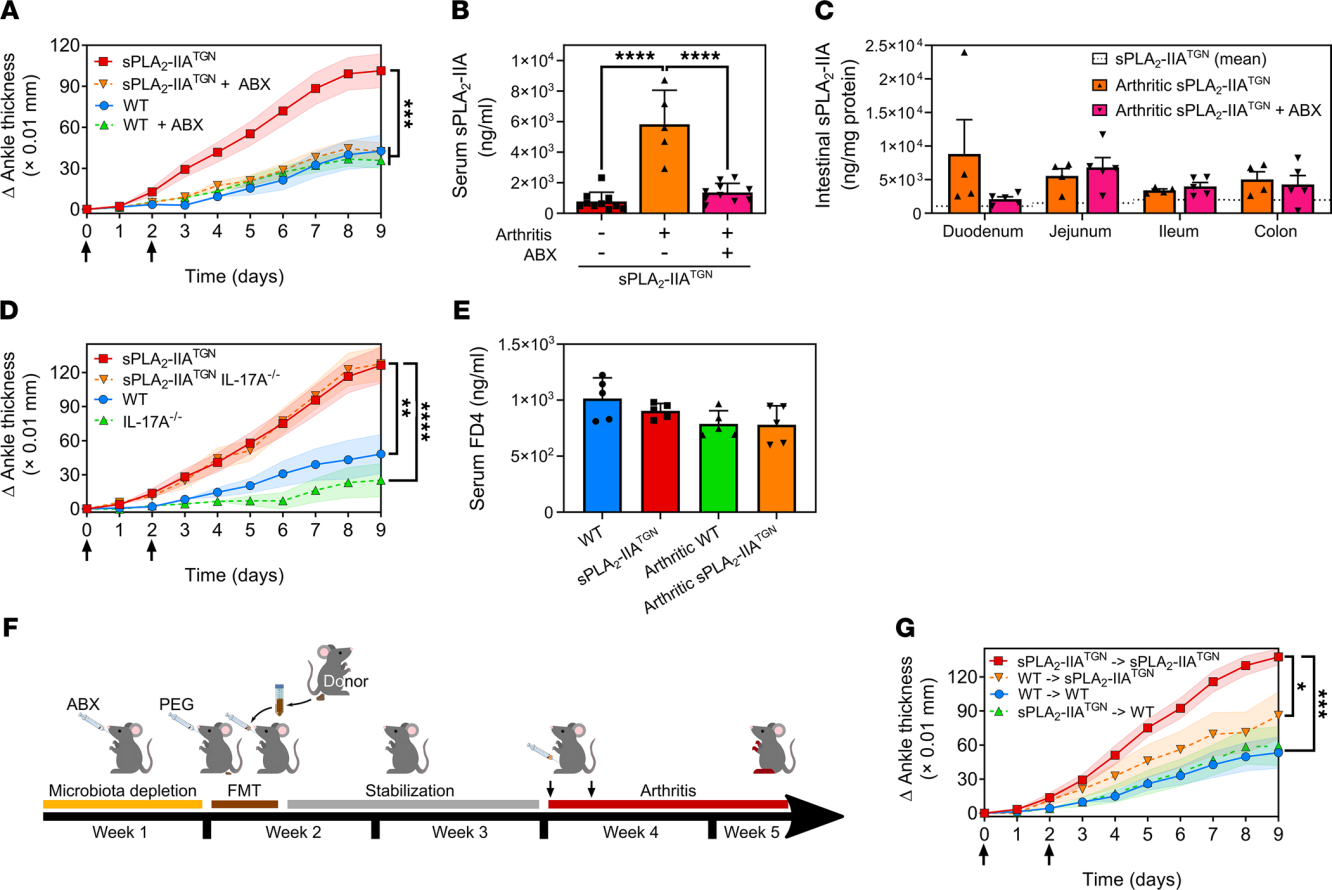
The sPLA_2_-IIA–mediated increased susceptibility to arthritis is dependent upon the intestinal flora. (**A**) Twelve-week-old WT and sPLA_2_-IIA^TGN^ mice housed in an Elite SPF+ animal facility were administered broad-spectrum antibiotics for 23 days. On experimental days 14 and 16, mice were injected i.p. with 150 μL of K/B×N serum (black arrows) to induce arthritis, and the disease severity was monitored daily by measuring ankle thickness (*n* = 16–30 from 2 separate experiments). (**B** and **C**) Quantification of sPLA_2_-IIA by time-resolved fluoroimmunoassay in serum (*n* = 5–10) and the intestinal compartments of arthritic sPLA_2_-IIA^TGN^ mice treated or not with antibiotics (*n* = 4–5). Dotted line indicates mean concentration in sPLA_2_-IIA^TGN^ mice. (**D**) The severity of serum-transferred arthritis was evaluated in 12-week-old transgenic mice depleted of IL-17A (IL-17A^−/−^) (*n* = 8–10). (**E**) Assessment of intestinal permeability in arthritic and nonarthritic mice by quantification of serum 4 kDa FITC-Dextran (FD4) translocated to the circulation following administration by oral gavage (*n* = 5). (**F** and **G**) Ten-week-old WT and sPLA_2_-IIA^TGN^ mice were administered antibiotics for 1 week to deplete their microbiota. On day 7, mice were administered a polyethylene-glycol–based laxative to empty their bowels, and a fecal microbiota transplantation (FMT) was performed. In brief, fresh fecal matter solution was administered by oral gavage to mice once a day for 3 consecutive days. Mice were then allowed to rest for 10 days before arthritis was induced by injection of K/B×N serum (black arrows). The severity of the disease was monitored daily (*n* = 10). Data from 1 (**C**–**G**) to 2 (**A** and **B**) separate experiments are presented as mean ± SEM. Statistical analysis included the following: (**A**, **D**, and **G**) repeated-measures 2-way ANOVA evaluating the statistical variation between groups. (**B**, **C**, and **E**) One-way ANOVA with Tukey’s multiple comparisons test. *P < 0.05, **P < 0.01, ***P < 0.001, ****P < 0.0001.

As the immune phenotype developed spontaneously over an extensive period of time, we hypothesized that the macroscopic observations made in these middle-aged mice were due to sustained action of sPLA_2_-IIA toward the microbiota but that a proinflammatory contribution of sPLA_2_-IIA through the involvement of the microbiota may be visible earlier if an inflammatory trigger was present. We therefore sought to subject mice to an immune challenge to further evaluate the functional impact of sPLA_2_-IIA–microbiota interactions on immunity. Given that young sPLA_2_-IIA^TGN^ mice are more susceptible to K/B×N serum–transferred arthritis (35, 86, 87) and that the microbiota can contribute to RA (88), we investigated the susceptibility to inflammatory arthritis of 12-week-old sPLA_2_-IIA^TGN^ mice either conventionally colonized or treated with antibiotics to deplete microbiota. While the depletion of the flora using ABX did not impact the development of arthritis in WT mice, it abolished the susceptibility of sPLA_2_-IIA^TGN^ mice to induced arthritis, as evaluated by measuring ankle thickening (Figure 5A). The concentration of sPLA_2_-IIA in the serum of arthritic sPLA_2_-IIA^TGN^ animals was greatly elevated in arthritic mice, while depletion of the microbiota abolished this increase (Figure 5B). However, neither arthritis nor the ABX treatment affected the intestinal levels of sPLA_2_-IIA (Figure 5C). Of note is that the transgenic suppression of IL-17A in sPLA_2_-IIA^TGN^ mice did not impact arthritis severity in this model (Figure 5D), and intestinal permeability did not appear modulated in sPLA_2_-IIA^TGN^ or arthritic mice (Figure 5E). Thus, the role of sPLA_2_-IIA in arthritis implicates the mouse flora independently of IL-17A. Moreover, while sPLA_2_-IIA expression by cells in the intestine is unlikely to explain the increased expression of circulating sPLA_2_-IIA in arthritis, the flora contributes to the enhanced levels of circulating sPLA_2_-IIA (Figure 5, B and C).

To evaluate the contribution of microbiota to RA susceptibility in sPLA_2_-IIA^TGN^ mice, we performed fecal microbiota transplantations (FMT) in microbiota-depleted 10-week-old mice (Figure 5F). WT mice transplanted with microbiota from WT mice developed mild arthritis, while sPLA_2_-IIA^TGN^ mice transplanted with microbiota from sPLA_2_-IIA^TGN^ mice developed severe arthritis (Figure 5G). Notably, while the transplantation of microbiota from sPLA_2_-IIA^TGN^ mice had no effect on the severity of arthritis in WT mice, transplantation of microbiota from WT mice to sPLA_2_-IIA^TGN^ mice reduced arthritis severity (Figure 5G). This suggests that components present in the WT microbiota could have protective effects toward arthritis.

### Impact of sPLA_2_-IIA and arthritis on intestinal microbiota composition.

**Figure 6:**
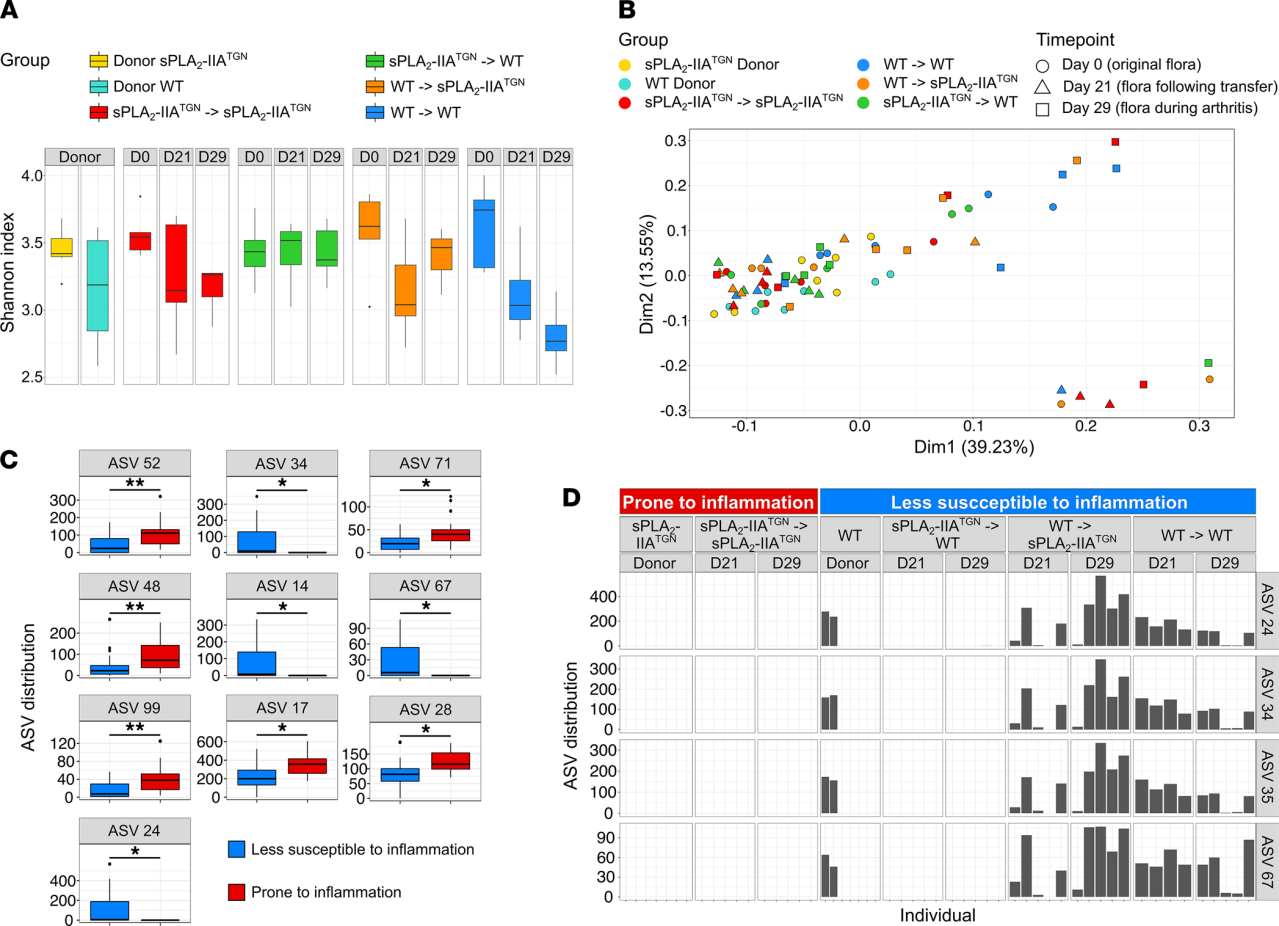
Mice with enhanced arthritis susceptibility present limited microbiota alterations. The fecal microbiota of donor and recipient mice involved in the fecal microbiota transplantation was sequenced at 3 different time points: before the transplantation (D0), 10 days following the transplantation (D21), and 8 days following the induction of arthritis (D29). (**A**) α-Diversity (Shannon index) of the microbiome of each mouse group at every time point (*n* = 5–7). (**B**) Principal component analysis based on the Bray Curtis dissimilarity comparing the flora from all mouse groups. (**C** and **D**) Groups were organized into 2 categories depending on their susceptibility to induced arthritis: sPLA_2_-IIA^TGN^ donors and sPLA_2_-IIA^TGN^ mice receiving the flora from sPLA_2_-IIA^TGN^ donors were classified as “Prone to inflammation,” and WT donors, WT recipients, and sPLA_2_-IIA^TGN^ mice receiving the WT flora were labeled “Less susceptible to inflammation.” (**C**) Distribution of amplicon sequence variants (ASV) differentially modulated between categories. (**D**) Distribution of differentially enriched ASVs found only in WT donors and their recipient mouse groups within every mouse. Bacterial species with highest percentage of identity for each differentially enriched ASV are as follows: ASV 52, *Ruminococcus bromii* (99%); ASV 48, *Vallitalea promyensis* (87%); ASV 99, *Anaerobacterium chartisolvens* (88%); ASVs 24, 34, and 35, uncultured *Muribaculum* species (100%); ASV 17, *Muribaculum intestinale* (91%); ASV 71, *Pseudoflafonifractor phocaeensis* (89%); ASV 67, Uncultured *Muribaculum* specie (96%); and ASV 28, *Muribaculum intestinale* (92 %). Data are presented as boxes representing the median and quartiles, with whiskers extending up to 1.5 interquartile range. Statistical analysis included Welch’s *t* test with *P* value corrected by Benjamini-Hochberg FDR procedure. Percentage of identity represent the percentage of similarity to the closest known sequence (Blastn using RefSeq NT). *P < 0.05, **P < 0.01.

To identify changes in microbiota following the FMT, DNA was extracted from feces at multiple time points (D0, original flora and flora from donors; D21, after transplantation; and D29, during arthritis), and 16S rRNA gene sequencing analysis was performed. The samples’ microbial diversity was compared using the QIIME2 pipeline, allowing for the discrimination of sequence variants at the nucleotide level (Amplicon Sequence Variant [ASV]). Analysis of α-diversity within each group failed to identify a trend following arthritis induction: the Shannon index remained unchanged in mice receiving the sPLA_2_-IIA^TGN^ flora, was slightly elevated in sPLA_2_-IIA^TGN^ mice receiving the WT flora, and was reduced in WT mice receiving the WT flora (Figure 6A). Surprisingly, despite the marked phenotype observed in sPLA_2_-IIA^TGN^ mice and the potent bactericidal activity of sPLA_2_-IIA, PCA based on the Bray-Curtis dissimilarity distance of the ASV distribution did not permit to highlight any clusters of samples (Figure 6B). Similar observations were also made in older sPLA_2_-IIA^TGN^ and WT mice housed in the Elite facility (Supplemental Figure 8), which is in stark opposition with prior observations made in the SPF animal facility. To identify more subtle alterations possibly involved in the amplification of arthritis, mice were separated into 2 distinct comparison groups: (a) prone to inflammation (sPLA_2_-IIA^TGN^ donors, and sPLA_2_-IIA^TGN^ mice recipient of the sPLA_2_-IIA^TGN^ flora) and (b) less susceptible to inflammation (WT donors, WT mice recipient of any flora, and sPLA_2_-IIA^TGN^ mice recipient of the WT flora). Following differential analysis between the 2 larger groups, we identified 6 ASVs that were more abundant in mice prone to inflammation (Figure 6C and Supplemental Table 2). Additionally, 4 ASVs were absent from donor sPLA_2_-IIA^TGN^ mice but were transferred from WT donors to sPLA_2_-IIA^TGN^ recipient mice (Figure 6, C and D, and Supplemental Table 2). Interestingly, these 4 ASVs were all associated with the same genus: *Muribaculum*. Three shared 100 % sequence identity with an uncultured bacterium of this genus, and the fourth, although predominantly associated with this genus, only shared a maximum of 96.45%. Thus, this points to a potential impact of bacteria associated with these ASVs in the protection against inflammation in WT mice.

### Role of sPLA_2_-IIA on gut lipidome.

**Figure 7:**
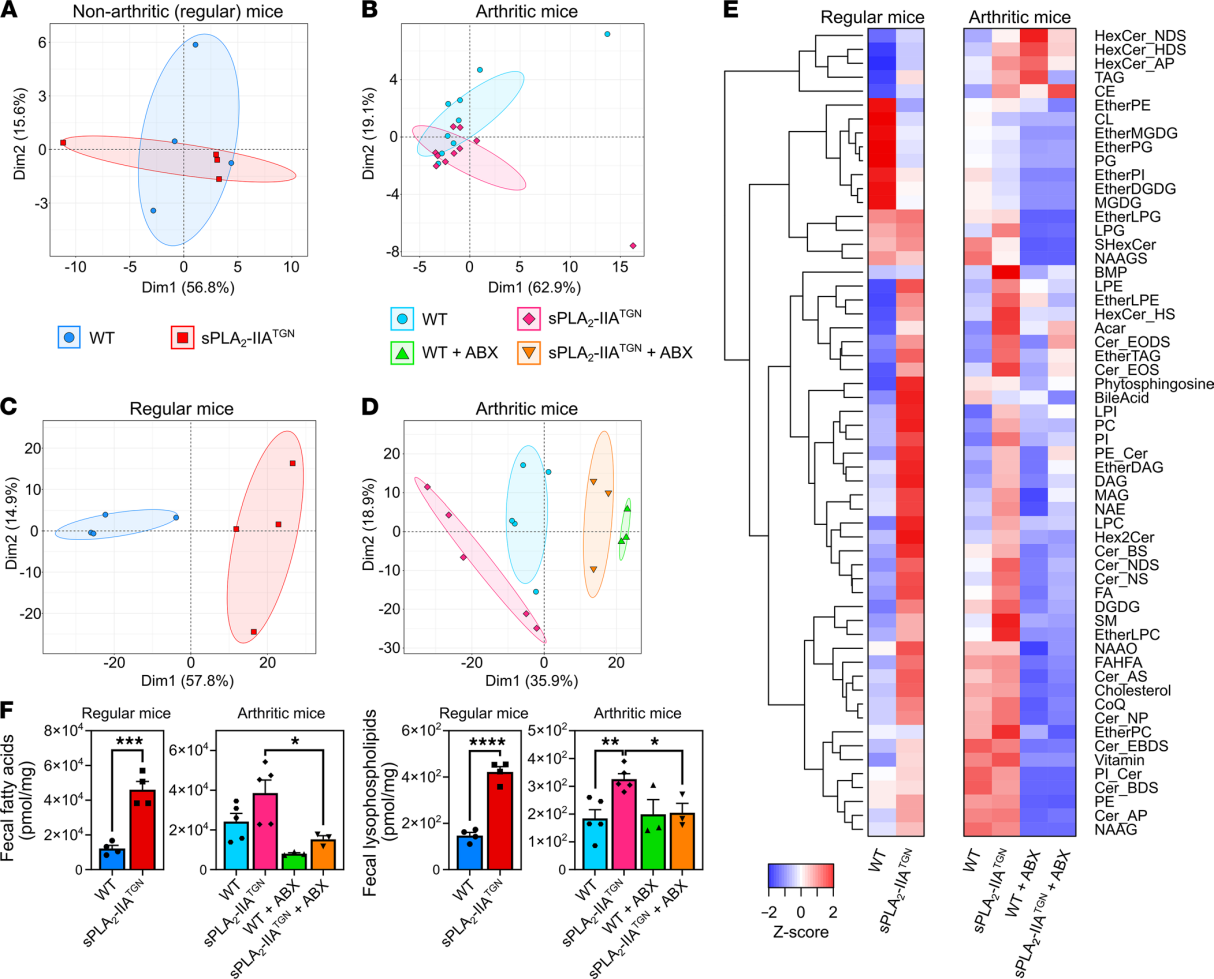
Mice expressing sPLA_2_-IIA possess an altered fecal lipidome. The intestinal and fecal lipid profile of 14-month-old and arthritic 14-week-old male mice housed in the Elite SPF+ animal facility was investigated. (**A** and **B**) Lipids were isolated from intestinal samples and identified using high-performance liquid chromatography combined with mass spectrometry. The data distributions for 14-month-old (*n* = 4) (**A**) and 14-week-old arthritic mice (*n* = 9–10) (**B**) were visualized by principal component analysis (PCA) with 99 % confidence ellipses. (**C**–**F**) An untargeted lipidomic analysis was performed using murine fecal samples. Data from 14-month-old (**C**) and 14-week-old arthritic mice (**D**) treated or not with antibiotics were visualized by PCA with 99 % confidence ellipses (*n* = 3–5). (**E**) Heatmap of the *Z* scores of the measured lipid classes for each experimental group. (**F**) Concentration of total fatty acids and lysophospholipids in samples from each mouse group (*n* = 3–5). (**F**) Data are presented as mean ± SEM. Statistical analysis included the following: unpaired *t* test and 1-way ANOVA with Šidák multiple comparisons test. *P < 0.05, **P < 0.01, ***P < 0.001, ****P < 0.0001.

The modest impact of sPLA_2_-IIA expression on microbiota composition in the Elite environment was in stark contrast with the marked proinflammatory phenotypes observed in sPLA_2_-IIA^TGN^ mice. Given that the primary function of sPLA_2_-IIA is to hydrolyze phospholipids, we investigated the enzyme’s contribution to eicosanoid production in the gut. Middle-aged (i.e., 14 months) and 14-week-old arthritic mice were included in the study. Examination of the tissue distribution of sPLA_2_-IIA in humans, using available public transcriptomic and proteomic analyses (89–91), reveals that the intestine is among the tissues expressing most sPLA_2_-IIA. Other PLA_2_s may be found in the intestine (92). However, the complete inhibition of sPLA_2_ activity on bacterial membranes in intestinal lysates from sPLA_2_-IIA^TGN^ mice by both EDTA and the specific inhibitor LY311727 suggested that this activity is mainly mediated by the group IIA sPLA_2_ (Supplemental Figure 7C). Targeted metabolomic analyses enabled identification and quantification of eicosanoids and a broad array of other endogenous lipid mediators in colon tissue samples of both WT and sPLA_2_-IIA^TGN^ mice (Supplemental Table 3). However, PCA revealed no clear clustering of samples among conditions, suggestive of limited metabolite changes (Figure 7, A and B). Only prostaglandin F_2α_ (PGF_2α_) and 1a,1b-dihomo-PGF_2α_ were modestly, but significantly, increased in sPLA_2_-IIA^TGN^ mice. Moreover, arthritic sPLA_2_-IIA^TGN^ mice displayed a significant reduction in N-docosahexaenoyl-ethanolamine (DHEA), palmitoylethanolamide (PEA), and 13-hydroxy-octadecadienoyl-glycerol (13-HODE-G) compared with arthritic WT mice of similar age. There was no overlap in the perturbed eicosanoids identified in sPLA_2_-IIA^TGN^ and arthritic mice.

Lipids derived from the microbiota, and notably short chain fatty acids, have immunomodulatory roles (93–95). Given the limited impact of sPLA_2_-IIA on the host’s gut eicosanoid profiles, we investigated its impact on bacterial lipids. Untargeted lipidomics were used to extensively characterize the lipid content of fecal samples from WT and sPLA_2_-IIA^TGN^ mice. sPLA_2_-IIA expression led to profound alterations in the fecal lipid profile (Figure 7, C and D, and Supplemental Table 4), as revealed by PCA of lipidomic data. Among the significantly modulated lipid subclasses, cholesteryl ester, ether-linked diacylglycerol (DAG), lysophosphatidylcholine, and triacylglycerol (TAG) — in addition to multiple lysophopholipids (lysophosphatidylcholine, -ethanolamine, and ‑inositol) and phospholipids (phosphatidylcholine and ‑inositol) — were elevated in both arthritic and nonarthritic sPLA_2_-IIA^TGN^ mice (Figure 7E). An increase in the concentration of total fatty acids and lysophospholipids was determined, which depended on sPLA_2_-IIA expression. This increase was observed in both nonarthritic and arthritic male sPLA_2_-IIA^TGN^ mice and was mitigated in the latter upon microbiota depletion by antibiotics (Figure 7F).

**Figure 8:**
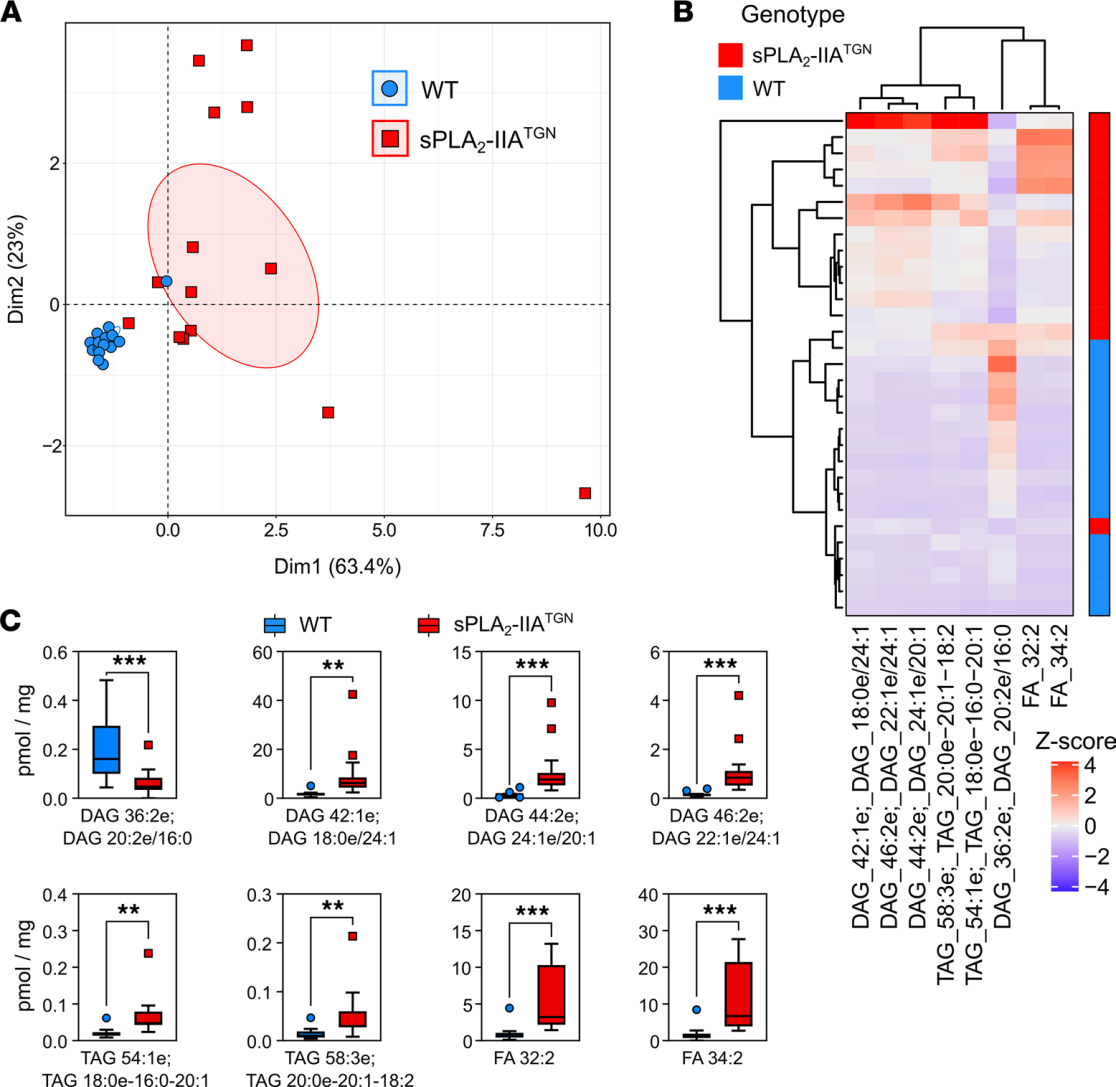
Identification of the expression of sPLA_2_-IIA using its fecal lipid signature. Machine learning was used to generate a fecal lipid signature able to distinguish WT and sPLA_2_-IIA^TGN^ mice independently of their housing facility and sex (*n* = 15–16 nonarthritic WT and sPLA_2_-IIA^TGN^ mice housed in either the SPF or Elite animal facility for 8 or 14 months, respectively). (**A**) Visualization of the data distribution using the identified lipids by PCA with 99% confidence ellipses to confirm the discrimination between the groups. (**B**) Heatmap of the *Z* scores — i.e., the number of SD above or below the mean, calculated from the concentration of the lipids. (**C**) Concentration of the 8 identified lipid metabolites in fecal samples. DAG, diacylglycerol; TAG, triacylglycerol; FA, fatty acid. Data are presented as boxes representing the median and quartiles, with whiskers extending up to 1.5 interquartile range. Statistical analysis included unpaired *t* test. **P < 0.01, ***P < 0.001.

Despite the fact that both male and female sPLA_2_-IIA^TGN^ mice developed spontaneous immune phenotype, the alterations were less prominent in the fecal lipidome of female Elite-housed and SPF-housed mice (Supplemental Figure 9). We thus used these clues to narrow the fecal lipid signature associated with sPLA_2_-IIA expression, independently of the housing facility and sex of the mice. Machine learning was used to identify whether the abundance of specific lipids could efficiently highlight sPLA_2_-IIA expression. BioDiscML is a machine learning sequential minimal optimization algorithm designed to identify predictive features within a given condition and was utilized as biomarker discovery tool (96). The use of BioDiscML permitted the identification of a discriminating lipid signature in sPLA_2_-IIA^TGN^ mice (Supplemental Figure 10). The signature comprised 8 lipids, namely DAGs, TAGs, and fatty acids, with a prediction accuracy of 100% on the training set and 91% on the testing set (Figure 8). The classification errors are believed to originate from 2 mice, 1 from each facility, that present similarities with the opposite group, as observed by PCA and heatmap clusters (Figure 8, A and B). The algorithm also reported 27 other lipids correlated with the 8-lipid signature (*r* < –0.99 or *r* > 0.99 or similar information gain) (Supplemental Figure 11). In addition to DAGs, TAGs, and fatty acids, the signature also included multiple ceramides, suggesting that members from these 4 families of lipids from the microbial lipidome suffice to discriminate between the 2 groups of mice.

## Discussion

sPLA_2_-IIA was historically named synovial sPLA_2_ and platelet-type sPLA_2_ given its original identification in the synovial fluid of RA patients and in platelets, respectively (27, 34). As such, overexpression of this enzyme during inflammatory processes and its ability to release arachidonic acid from membrane phospholipids stimulated extensive research into the relevance of this activity in patients suffering from chronic inflammatory diseases (28–33). It took decades to appreciate the enzyme’s bactericidal properties, which pointed toward its belonging to the host antibacterial arsenal (40, 97–99). However, despite dominant expression in the intestine and potent bactericidal activity (34, 43), whether or how the antimicrobial properties of sPLA_2_-IIA impact the intestinal flora during inflammatory disorders remained poorly documented. In the present study, we demonstrate that the interplay between sPLA_2_-IIA and the microbiota profoundly affects the immune system, contributing to different immune phenotypes.

The overexpression of sPLA_2_-IIA spontaneously led to an immune phenotype characterized by enlarged MDLNs, likely due to accumulation or sequestration of leukocytes in these organs. The loss of immune homeostasis became evident in aging mice between 28 and 40 weeks of age. Other lymph nodes (ILN, PLN) and the spleen were also enlarged, which suggested that expression of sPLA_2_-IIA has systemic immune consequences. Moreover, the proportion of granulocytes increased while that of lymphocytes decreased in both the BM and spleen of sPLA_2_-IIA^TGN^ mice. These reactions may be reminiscent of a myeloid shift in the BM and the presence of systemic inflammation. Further characterization would, however, be required to confirm this shift. This is nonetheless supported by elevated levels of circulating IgG and IgA, which are consistent with the increased number of plasmablasts in MDLNs and may implicate the microbiota (100–102). Despite the elevated circulating concentration of IL-17A, the genetic ablation of this cytokine did not prevent the development of the immune phenotype.

Housing conditions could affect sPLA_2_-mediated immune phenotype, which was not attributable to lower levels of circulating sPLA_2_-IIA in a facility with higher standards. The catalytic activity of circulating sPLA_2_-IIA also remained unchanged between facilities, but it is not excluded that changes in the activity of intestinal sPLA_2_ may be involved. Moreover, transgenic expression of sPLA_2_-IIA did not affect the expression of other AMPs by Paneth cells, as quantitative PCR (qPCR) revealed no significant modulation in the relative expression of α-Defensin, RegIIIβ, RegIIIγ, and lysozyme C in ileum samples from 14-month-old sPLA_2_-IIA^TGN^ and WT mice housed in the Elite facility (Supplemental Figure 3F). Since the main differences between animal facilities aim to further decrease the risks of contamination and infection by opportunistic pathogens, the higher standards are likely to have a favorable impact on the intestinal flora of housed animals, supporting its potential implication in the sPLA_2_-IIA–mediated immune phenotype. The combination of sPLA_2_-IIA with a certain microbiota composition, and the fact that the immune phenotype becomes noticeable only in relatively aged mice, may also explain how this spontaneous immune phenotype had yet to be characterized despite the development of the sPLA_2_-IIA^TGN^ mouse model more than 20 years ago (103). Despite tremendous efforts, we were unable to generate germ-free sPLA_2_-IIA^TGN^ mice. However, near-complete (>99%) depletion of the intestinal flora using a broad-spectrum antibiotic treatment significantly reduced multiple aspects of the phenotype, thereby confirming that this immune process found in sPLA_2_-IIA^TGN^ mice is microbiota dependent and can be reversed.

Depletion of the intestinal flora also abolished the increased susceptibility to induced arthritis in approximately 12-week-old sPLA_2_-IIA^TGN^ mice. This is not the first finding that points to a role of the microbiota in this arthritis model, as the segmented filamentous bacteria (SFB) was found to promote the release of IL-17A in K/B×N mice: the same mice that are utilized as a source of arthritogenic serum (104–106). However, in sPLA_2_-IIA^TGN^ mice, the genetic ablation of IL-17A failed to reduce the severity of arthritis. This suggests that IL-17A is dispensable for the enhanced arthritis observed in sPLA_2_-IIA–expressing mice using the K/B×N serum-transfer model. sPLA_2_-IIA was suggested to contribute to sterile inflammation through its activity on membranes from apoptotic cells (107) or extracellular vesicles (85). Although the exact mechanism remains to be identified, this study demonstrates the functional association between the microbiota and sPLA_2_-IIA in the promotion of inflammatory arthritis,

Blood levels of sPLA_2_-IIA are elevated in atherosclerosis and RA (108, 109). The enzyme expression is also increased in the gut of individuals with Crohn’s disease (30). While the levels of sPLA_2_-IIA measured in blood (250–2300 ng/mL [up to 8700 ng/mL]) of sPLA_2_-IIA^TGN^ mice are elevated in comparison with those measured in healthy individuals (64), these levels remain comparable with those measured in patients affected by bacterial infections and sepsis (28, 110). The use of sPLA_2_-IIA^TGN^ mice has, therefore, permitted an insight into immune manifestations that might occur in humans affected with these chronic diseases. These observations are relevant, as human sPLA_2_-IIA was found in mouse intestine, consistent with its expression in humans. Moreover, it cannot be excluded that other members of the sPLA_2_ family may be implicated in the modulation of the immune system in mice, and that these contributions may be masked, given the overexpression of human sPLA_2_-IIA in our model. As an example, the expression of sPLA_2_-IIF has been documented in the skin of both humans and mice (111), and although it does not possess bactericidal properties (99), it may interact with skin flora.

Bacterial stimuli can promote the expression of sPLA_2_-IIA (70–74). Therefore, we initially hypothesized that sPLA_2_-IIA expression in the intestine was under the control of the intestinal flora, which in turn could regulate immune homeostasis or arthritis severity. However, circulatory and intestinal levels of sPLA_2_-IIA in addition to its enzymatic activity in serum remained unchanged upon housing mice in different environments and following microbiota depletion. Moreover, although intestinal permeability may be increased in inflammatory conditions (112), thereby providing a connection between factors from the microbiota and the circulatory system, we failed to observe any significant impact of sPLA_2_-IIA on this process. While the transgene-driven sPLA_2_-IIA expression may explain the constant sPLA_2_-IIA expression in the intestine, it is interesting to point out that sPLA_2_-IIA was greatly elevated in blood in arthritic conditions but remained unchanged in the intestine. Furthermore, depletion of microbiota abrogated the elevation of sPLA_2_-IIA in blood, suggesting that sPLA_2_-IIA expression can be induced even in a transgenic model. Together, this suggests that the reduced inflammatory phenotypes in mice housed under Elite conditions and in microbiota-depleted mice are unlikely to be due to modulation of sPLA_2_-IIA in the intestine. Elevated counts of circulating leukocytes were previously observed in sPLA_2_-IIA^TGN^ mice in response to LPS (113). It would be plausible that inflammatory stimuli such as TNF-α, IL-1, and IL-6, all induced during K/B×N serum–transferred arthritis (114), promote sPLA_2_-IIA expression in these leukocytes, leading to increased circulating sPLA_2_-IIA concentrations in this model. As infectious stimuli such as LPS and other PAMPs may also induce sPLA_2_-IIA expression (70, 113), the elimination of the intestinal flora using antibiotics could suppress this stimulation, leading to the lower concentration found in arthritic mice treated with antibiotics. Bacterial extracellular vesicles, which bear PAMPs and bacterial membranes, are described in the blood circulation in humans and can interact with immune cells (115, 116). It is possible that these vesicles induce systemic expression of sPLA_2_-IIA and also act as an ideal substrate for sPLA_2_-IIA, for which few endogenous substrates are known (34). The source of circulating sPLA_2_-IIA in arthritic conditions and how the microbiota affects its release remain to be explored.

Transplantation of the flora from sPLA_2_-IIA–expressing mice to WT mice did not impact the severity of arthritis. Thus, proarthritic factors are not transplantable or may not suffice to promote arthritis by themselves. We therefore speculate that the local release of lipid mediators from bacteria by sPLA_2_-IIA in the gut, rather than the presence of proinflammatory bacterial strains, may enhance arthritis and inflammation. A similar mechanism has previously been proposed, as infection of sPLA_2_-IIA^TGN^ mice was shown to also promote neutrophilia, possibly through the liberation of prostaglandins (113). Neutrophils play a key role in the inflammatory response to K/B×N serum–transferred arthritis (117, 118), and their abundance in sPLA_2_-IIA^TGN^ mice may contribute to the enhanced arthritis found in these animals. The recruitment of immune cells due to the activity of sPLA_2_-IIA toward bacterial membranes may also represent another mechanism by which the enzyme contributes to the defense against bacterial infections. Whether or how the microbiota contributes to the neutrophil response in transgenic mice will need further investigation.

Transplantation of the WT flora into transgenic mice led to a decrease in the severity of arthritis, suggesting that WT flora may play a protective role in this process. While the exact mechanism responsible for this decrease remains unclear, the only ASVs found more abundantly in mice less susceptible to inflammation appeared associated with uncultured bacteria from the *Muribaculum* genus. To this day, *Muribaculum intestinale* is the sole cultured bacterium within this genus. As this bacterium has only recently been described in mice (119), little is known regarding its interactions with the immune system. However, since this genus was not found in all WT donor mice and in none of the WT mice transplanted with the sPLA_2_-IIA^TGN^ flora, it cannot be solely accountable for their resistance to arthritis in absence of sPLA_2_-IIA. Furthermore, a greater impact of the intestinal flora on the amplification of inflammation is not excluded, as specific bacteria are known to promote arthritis (104).

Our mechanistic investigations were mostly carried out using mice housed in an Elite animal facility. While these standards should allow for a greater reproducibility of the investigations performed in facilities with similar high standards, the decision to concentrate our efforts using the Elite facility was also due to the unavailability of our SPF facility at the later stages of the study. As opposed to the Elite facility, pronounced alterations of the intestinal microbiota were observed in sPLA_2_-IIA^TGN^ mice housed in the SPF animal facility. Out of the 10 most abundantly and differentially enriched genera, all of the Gram-negative genera were more abundant in sPLA_2_-IIA^TGN^ mice and all but 1 Gram-positive genera (*Lactobacillus*) were more abundant in WT mice, which is consistent with the preference of sPLA_2_-IIA for Gram-positive bacteria. Notable bacteria found at elevated abundances in sPLA_2_-IIA–expressing mice include *Helicobacter* and *Prevotella* species, both previously associated with inflammatory processes (120–123). However, as mice still developed immune disturbances in the Elite facility, the alterations in bacterial populations observed in the SPF facility do not appear solely responsible for the development of this phenotype. Nevertheless, the composition of the fecal flora was drastically different in mice housed in the Elite compared with the SPF facility, being respectively dominated by bacteria from the *Firmicutes* or *Bacteroidetes* phyla. These compositional differences, in addition to the loss of inflammation-associated bacteria such as *Helicobacter* and *Prevotella* in Elite-housed mice, could explain the enhanced severity of the immune phenotype in mice housed in the SPF facility. While the loss of the marked alterations in the microbiota composition of mice housed in the Elite facility impeded further mechanistic investigation of the role played by these bacteria in the immune phenotype, the study design nonetheless permitted to suggest that lipids from the microbiota, rather than the microbiota composition itself, may regulate inflammation.

We report modest changes to the colonic eicosanoid profile due to sPLA_2_-IIA expression. PGF_2α_ was increased in the colon of sPLA_2_-IIA^TGN^ mice and has been shown to be abundant at inflammation sites and, in particular, in the synovial fluid of RA patients (124). Therefore, this warrants further investigation into a possible role for this mediator in the immune phenotype of sPLA_2_-IIA^TGN^ mice. However, this metabolite was not significantly increased in arthritic sPLA_2_-IIA^TGN^ mice. Instead, reduced levels of DHEA, PEA, and 13-HODE-G were found. It was previously demonstrated that both DHEA and PEA may attenuate inflammation (125–127). Therefore, their reduction in mice displaying an increased inflammatory arthritis severity may highlight a possible protective role for these metabolites in this process. 13-HODE-G was recently shown to be synthesized by eosinophils and neutrophils, but its biological functions remain unknown (128). sPLA_2_-IIA can release arachidonic acid through its activity on phospholipid membranes for eicosanoid production (129). Thus, the absence of alterations in the arachidonic acid content of samples from sPLA_2_-IIA^TGN^ mice was unsuspected. This, in addition to the limited modulation in the eicosanoid content in intestinal samples, suggests that — contrary to our initial hypothesis — sPLA_2_-IIA may not contribute to inflammation through the production of eicosanoids in the intestine in this model. The fact that both cPLA_2_-α and 12-LO, which may contribute to the production of eicosanoids in collaboration with sPLA_2_-IIA (33, 81–85), had no impact on the immune phenotype also supports these observations.

Bee and wasp venom sPLA_2_s can generate neoantigens from phosphodiacylglycerides, thereby stimulating CD1a NKT cell (130). It is, therefore, possible that sPLA_2_-IIA might activate these immune cells through release of lipid metabolites from bacterial membranes. Supporting this, profound alterations in the fecal lipidome of arthritic and nonarthritic sPLA_2_-IIA^TGN^ mice housed in the Elite environment were measured. Using machine learning, we suggest that metabolites belonging to the DAG, TAG, fatty acid, and ceramide families of lipids are associated with sPLA_2_-IIA expression. While most of the identified lipids were more abundant in sPLA_2_-IIA^TGN^ mice, 1 DAG highlighted in the lipid signatures was more abundant in WT mice. It remains to be explored whether specific lipids from these families may play divergent roles. Whether lipids released by sPLA_2_-IIA from bacterial membranes can serve as antigens for NKT cells is also an appealing direction of research (131, 132). In sum, our work further highlights that, in addition to maintenance of the microbiota diversity, a fine balance of the microbiota lipidome may have important repercussions on health.

Interestingly, similar overall observations were concurrently made by Miki et al. and can be found in the accompanying manuscript (133). Using a KO model of sPLA_2_-IIA in the BALB/c background, they notably demonstrated that the expression of sPLA_2_-IIA impacts skin carcinogenesis and psoriasis through: the alteration of the microbiome; the modulation of the expression profile of genes implicated in immunity, epithelial barrier, and metabolism; and the induction of changes to the fecal lipidome. Consistent with our observations, bacteria from the *Prevotella* genus appeared more abundant in mice expressing the enzyme in this study. However, the results in both studies are not fully aligned, as Miki et al. observed elevated abundances of *Helicobacter* and *Ruminococcus* genera in mice devoid of sPLA_2_-IIA. The use of different sPLA_2_-IIA expression models with different genetic background and housed in different environments may explain the discrepancies between the studies. Nevertheless, the reduction of the immune phenotypes upon cohousing the mice expressing or not sPLA_2_-IIA is consistent with the reduced arthritic phenotype of sPLA_2_-IIA^TGN^ mice transplanted with the WT flora. Additionally, they also observed an important impact of the housing condition upon transferring mice to another animal facility. Therefore, with the use of a completely different approach, Miki et al. reach similar conclusions as to the ones presented in this article: sPLA_2_-IIA produces systemic perturbations through its activity on the intestinal microbiota and related lipidome.

AMPs can directly affect bacterial viability and may, as such, contribute to dysbiosis (19). Other enzymes that metabolize amino acids, such as arginase, can also impact the integrity of the intestine endothelium and its microbiota (134). The findings reported herein identify a singular protein, the sPLA_2_-IIA, as an enzyme that contributes to sterile inflammatory conditions through functional interactions with the microbiota and associated lipidome. This adds sPLA_2_-IIA to the list of endogenous factors capable of affecting the immune system and microbe-host homeostasis.

## Methods

Supplemental Methods are available online with this article.

### Data availability.

Sequence data generated during the study that support the findings have been deposited in Gene Expression Omnibus (GEO) with the accession no. GSE189441 (Figure 2, Figure 6, and Supplemental Figure 8).

### Statistics.

Statistical analyses were performed using GraphPad Prism version 9 (GraphPad Software) or R v3.2.2 (135) for the analysis of sequencing and lipidomic data. Measurements were taken from distinct biological samples, with the exception of arthritis severity assessment; in that case, severity was measured daily on each mouse by an investigator blinded to treatment groups. Statistical tests and associated corrections are reported at the end of each figure. Two-tailed unpaired *t* test and 1-way ANOVA were used to compare 2 groups or more, respectively. Two-way ANOVA was used for analysis of repeated measures. For larger data sets, Welch’s *t* test and Wald test were used with a *P* value corrected by Benjamini-Hochberg FDR procedure. *P* values of 0.05 and less were considered significant for the purpose of this study. *P < 0.05, **P < 0.01, ***P < 0.001, ****P < 0.0001.

### Study approval.

All animal protocols were approved by the Animal Welfare Committee at CHU de Québec-Université Laval (no. 17-142), and the study followed the Guidelines of the Canadian Council on Animal Care.

## Author contributions

EB and ED designed the study and prepared the manuscript; CJB, AM, AB, and AD performed the microbiome analysis; SM, MA, CM, and NF performed the lipidomic analysis; ML performed machine learning analysis; TL, IA, ACD, and NC contributed to the characterization of the immune phenotype; CP, APP, and GL quantified sPLA_2_-IIA; MHG generated cPLA2-α^−/−^ mice; and MR, MM, and LD provided critical insight for the design of some experiments.

## Supplementary Material

Supplemental data

Supplemental data set 1

Supplemental table 2

Supplemental table 3

Supplemental table 4

Supplemental table 6

## Version 1 01/25/2022

Electronic publication
