# Supplementary material for: The interaction of secreted phospholipase A2-IIA with the microbiota alters its lipidome and promotes inflammation
Source: JCI Insight. 2022 Jan 25;7(2):e152638. doi: 10.1172/jci.insight.152638 (PMC8855825; doi:10.1172/jci.insight.152638)
Supplement: Supplemental data [file jciinsight-7-152638-s155.pdf]

Supplemental methods

Mice

Our study followed the Guidelines of the Canadian Council on Animal Care and the protocols were approved by the Animal Welfare Committee at CHU de Québec-Université Laval. C57BL/6J (WT), *Alox12*$^{-/-}$ and *Il17a*$^{tm1.1(icre)Stck}$/J (IL-17A$^{-/-}$) mice were obtained from The Jackson Laboratory, and the cytosolic phospholipase A_2-α-deficient mice (cPLA$_2^{-/-}$) (1) and mice overexpressing human sPLA$_2$-IIA (sPLA$_2$-IIA^{TGN}) (2, 3) have previously been described.

To obtain IL-17A$^{-/-}$ mice overexpressing human sPLA$_2$-IIA and given that sPLA$_2$-IIA^{TGN} females present deficient pup nurturing, sPLA$_2$-IIA^{TGN} males (2, 3) were crossed with IL-17A$^{-/-}$ females. The resulting sPLA$_2$-IIA^{TGN}::IL-17A$^{+/-}$ and sPLA$_2$-IIA$^{-/-}$::IL-17A$^{+/-}$ mice were then crossed to generate sPLA$_2$-IIA$^{-/-}$::IL-17A$^{+/+}$, sPLA$_2$-IIA$^{+/-}$::IL-17A$^{+/+}$, sPLA$_2$-IIA$^{-/-}$::IL-17A$^{-/-}$ (IL-17A$^{-/-}$) and sPLA$_2$-IIA$^{+/-}$::IL-17A$^{-/-}$ (sPLA$_2$-IIA^{TGN}::IL-17A$^{-/-}$) mice. sPLA$_2$-IIA^{TGN}::cPLA$_2^{-/-}$ and sPLA$_2$-IIA^{TGN}::*Alox12*$^{-/-}$ mice were obtained as previously described (4, 5). Mice were housed in a specific pathogen-free (SPF) or Elite SPF+ (Elite) animal facility at CHU de Québec-Université Laval, Quebec City, Canada.

Flow cytometry

Cells from mandibular lymph nodes (MDLN) and spleens were isolated by pressing organs through a 40-µm filter in PBS 1×. Bone marrow cells were isolated by flushing murine tibia and femur with PBS 1×. The flow-through was then passed through a 40-µm filter. The number of cells in the cell suspensions was determined using an automated cell counter (Nexcelom Bioscience). Flow cytometry was performed using a BD FACSCanto II instrument (BD Biosciences) (**Supplemental Figure 12**). In brief, 1×10^6 MDLN cells and 5×10^6 spleen and bone marrow cells were stained for 30 minutes in the dark before addition of fixation solution (4% paraformaldehyde (PFA)). Multiple fluorochrome-coupled antibodies were used: αCD3-Pacific blue clone 500A2, αCD19-PE-Cy7 clone 1D3, αGr1-

FITC clone RB6-8C5, αTer119-APC clone TER-119, αCD138-Bv421 clone 281-2, αB220-PE clone RA3-6B2 and αNK1.1-PercP clone PK136, all from BD Biosciences. Cells per ml were determined by adding a known concentration of 15 μm Polybead Microspheres (Polysciences, Inc) to each sample before flow cytometry acquisition. All results were analysed using FlowJo v10 software (Tree Star).

Complete blood count

Blood was collected from mice upon euthanasia by cardiac puncture and mixed with 0.2 volumes anticoagulant-citrate-dextrose (ACD) solution pH 4.5 (45 mM sodium citrate, 25 mM citric acid and 82 mM dextrose) and 0.35 volumes of Tyrode's solution pH 6.5 (135 mM NaCl, 3 mM KCl, 0.34 mM Na_2HPO_4, 12 mM $NaHCO_3$, 20 mM HEPES, 4.5 mM glucose, 0.5 mM $MgCl_2$ and 0.05% bovine serum albumin). To detect any disturbance in levels of circulating leukocytes, erythrocytes and platelets, a complete blood count was performed using a blood hematology analyser (scil animal care company).

Cytokine dosage

Cytokines were quantified using the Cytometric Bead Array (CBA) Mouse Enhanced Sensitivity system using the following Mouse Enhanced Sensitivity Flex Sets: IL-2, IL-6, IL-10, TNF-α, IL-17A and IL-1β, all from BD Biosciences. The recommended protocol was followed at half volume. Samples were acquired on a BD FACSCanto II and analysed using FCAP Array Software (version 3.0). Cytokines were also quantified in the serum of mice by Eve Technologies Corp using the Mouse High Sensitivity T-Cell Discovery Array 18-plex (MDHSTC18).

Immunoglobulin dosage

Immunoglobulin G and A were quantified using the IgG (Total) Mouse Uncoated ELISA Kit and IgA Mouse Uncoated ELISA Kit (Invitrogen), respectively. The recommended assay was performed at half volume using half area polystyrene microplates (Corning).

Phenotype scoring

Every second week, scores from 0 to 3 were attributed to $sPLA_2$-IIA^{TGN} mice housed in the Elite SPF+ animal facility according to the degree of swelling and the number and size of palpable nodes in the neck.

The mouse neck was closely examined to evaluate the presence of swelling. A swelling score of 0 was given to mice with no noticeable swelling. When mice presented a perceptible, but minor swelling, a score of 1 was attributed. Mice were given a score of 2 when the swelling became large enough to be easily perceptible. A score of 3 was attributed to mice presenting marked swelling that was extensively distributed or spread around the neck.

The mouse neck was also palpated to identify the presence of defined lumps. A lump score of 0 was attributed if none were detected. A score of 1 was given when a single and small solid lump was detected in the mouse neck. A score of 2 was awarded when multiple small to medium-sized solid lumps were detected by palpation. Mice with large lumps (> 1 cm) were attributed a score of 3.

Lysis of intestinal samples for $sPLA_2$-IIA activity and concentration assessment

Frozen intestinal samples were added to a 2 ml Lysing Matrix D tube (MP Biomedicals) containing 500 µl 0.1 M HCl, 1 M NaCl, 10 mM EDTA and 1× cOmplete™ protease inhibitor cocktail (Roche). Samples were vortexed vigorously in a FastPrep FP120 homogenizer (Thermo Savant) at a power level of 6.0 for 40 seconds. The resulting homogenate was centrifuged at 14,000 RPM for 15 minutes at 4°C to eliminate the beads and debris, and the supernatant was collected for $sPLA_2$-IIA quantification and enzymatic activity assessment.

Enzymatic activity

The enzymatic activity of $sPLA_2$-IIA was determined by incubating serum or intestinal lysates with *E. coli* membranes radiolabeled with [^{3}H]oleic acid as previously described (6). In brief,

30,000 dpm of radiolabeled *E. coli* membranes in activity buffer (0.1 M Tris-HCl pH 8.0, 10 mM $CaCl_2$, and 0.1% BSA) were incubated with recombinant human $sPLA_2$-IIA (1 to 50 pM final concentration), serum samples or intestine lysates at 37 °C for 1 hour. Enzymatic reactions were stopped by addition of 1 volume of 0.1 M EDTA, 0.2% fatty acid-free BSA. Tubes were centrifuged at 14,000 RPM and supernatant was collected and counted in a Tri-Carb liquid scintillation counter (PerkinElmer). To confirm the $sPLA_2$ and $sPLA_2$-IIA-specific activity, selected samples were respectively incubated with either 40 mM EDTA, which inhibits $sPLA_2$ activity, or 10 µM LY311727, a specific inhibitor of $sPLA_2$-IIA, at least 30 minutes before incubation with bacterial membranes.

Time-resolved fluorescence immunoassay

To quantify $sPLA_2$-IIA in serum and intestinal samples, 1 µg polymeric IgG targeting human $sPLA_2$-IIA was fixed overnight to 96-well Delfia Microtiter Plates (PerkinElmer Wallac, Turku, Finland) in 100 µl 0.1 M NaH_2PO_4 pH 4.9 at 4°C in a humidified atmosphere. The coating solution was then eliminated, and the plates were blocked for 2 hours on a plate agitator with 200 µl of filtered 0.9% NaCl, 50 mM Tris-HCl pH 7.8, 0.05% NaN_3, 6% D-sorbitol, 1% BSA, 1 mM $CaCl_2$ per well. The plates were washed with 200 µl wash solution prepared from DELFIA Wash Concentrate (PerkinElmer) and then 100 µl of samples diluted in assay buffer (140 mM NaCl, 50 mM Tris-HCl pH 7.8, 0.5% BSA, 0.05% NaN_3, 0.01% Tween 40, 20 µM DTPA) were added to each well. Samples were incubated for 2 hours with agitation then the plates were washed three times. One hundred microliters of polymeric human $sPLA_2$-IIA-targeting IgG stained with europium using the DELFIA Eu-Labeling kit (PerkinElmer) were added to each well. After an incubation of 1 hour 15 minutes with agitation, the plates were washed an additional three times and 100 µl of enhancing solution was added to chelate the europium. The plates underwent a final 5-minute incubation before detection using an EnVision TRF plate reader (PerkinElmer).

Microbiota depletion

Twelve-month-old sPLA$_2$-IIA^{TGN} and WT mice were administered ampicillin (1 g/L) in drinking water for 6 weeks. In addition, 200 µl of a neomycin sulfate (10 mg/ml), metronidazole (10 mg/ml), and vancomycin (5 mg/ml) cocktail, all from Cayman Chemical, was administered via oral gavage twice daily (7). Fresh antibiotics were prepared every second day, and water was administered as a control. For microbiota depletion assessment, stool samples were collected every second week, frozen immediately on dry ice and stored at −80°C until further analysis. Mice were euthanized six weeks following the first antibiotic administration. Blood was collected for serum and plasma preparation, and the spleen, MDLNs and bone marrow were harvested for analysis by flow cytometry. The intestine was excised, and the contents were collected and frozen on dry ice. The intestinal tissue was washed in PBS 1×, immediately frozen and stored at −80°C for future sPLA$_2$-IIA quantification.

Microbiota depletion assessment

DNA was extracted from stool samples using the DNeasy PowerSoil Kit (Qiagen) and quantified using a BioDrop µLite (Isogen Life Science). Quantitative PCR (qPCR) was performed using SsoAdvanced Universal SYBR Green Supermix (Bio-Rad). Primers targeting variable region 6 (V6) of the bacterial 16S ribosomal RNA gene and the mouse polymeric immunoglobulin receptor (m-pIgR) were used (**Supplemental Table 5**) (7). All reactions were performed in a total volume of 10 µl in a Rotor-Gene 3000 (Corbett Research). For 16S rRNA V6, 0.25 ng of DNA, or 25 ng of DNA for antibiotic-treated animals, was amplified and thermal cycles were set to 3 minutes at 98°C followed by 40 cycles comprising a denaturation step: 15 seconds at 98°C and an annealing and extension step: 15 seconds at 60°C. A dissociation curve was then produced to confirm amplification of the correct product by increasing the temperature by 0.5°C every 5 seconds from 65°C to 98°C. For m-pIgR amplification, 100 ng of DNA, or 25 ng for antibiotic-treated mice, were used. Thermal cycles were as for 16S rRNA V6, but the annealing/extension step was 15 seconds at 61°C. DNA concentration was

determined using a standard curve of known concentration generated with gBlocks gene fragment (Integrated DNA Technologies) designed to contain the sequences amplified by both primer pairs (**Supplemental Figure 13**). The number of copies was calculated by estimating the mass of a base pair of double stranded DNA at 660 g/mol.

Antimicrobial peptide expression assessment

Snap-frozen ileum tissue samples from WT and $sPLA_2$-IIA^{TGN} mice housed in the Elite animal facility for 14 months were homogenized in lysis buffer and RNA was isolated using the Aurum Total RNA Mini Kit (Bio-Rad) according to the manufacturer's protocol. RNA was transcribed using the iScript cDNA Synthesis Kit (Bio-Rad) and qPCRs were carried out in a Rotor-Gene Q (Qiagen) in a total volume of 10 µl. Primers targeting murine genes coding for antimicrobial peptides α-Defensin (*Defa1*), RegIIIβ (*Reg3b*), RegIIIγ (*Reg3g*) and lysozyme C (*Lyz1*), and housekeeping genes β-actin (*Actb*), hypoxanthine guanine phosphoribosyl transferase (*Hprt*) and 18S rRNA were used (**Supplemental Table 5**). Thermal cycles were set to 3 minutes at 95°C followed by 40 cycles of either 95°C for 15 seconds and 60°C for 30 seconds (*Actb*, *Defa1* and *Lyz1*) or 95°C for 30 seconds, 58°C for 30 seconds and 72°C for 30 seconds (18S rRNA, *Hprt*, *Reg3b* and *Reg3g*). Thermal cycling was followed by a dissociation curve as mentioned above. Relative abundance was evaluated using the $2^{-\Delta Ct}$ method where ΔCt was calculated by subtracting the average Ct of the three housekeeping genes (β-actin, 18S rRNA and HPRT) from the Ct of AMPs.

K/B×N serum-transferred arthritis

Arthritis was induced in male mice by intraperitoneal injections of arthritogenic K/B×N serum (150 µl) performed on experimental days 0 and 2. Ankle thickening, which correlates with disease severity, was monitored daily by measuring the malleoli using spring-loaded dial calipers (Newman Tools Inc.) with the ankle in a fully flexed position as previously described (8).

Fecal microbiota transplantation (FMT)

10-week-old male mice were administered ampicillin (1 g/l) in their drinking water and neomycin (10 mg/ml), metronidazole (10 mg/ml) and vancomycin (5 mg/ml) via oral gavage twice daily for 1 week. Twenty-four hours following the last antibiotic administration, mice were transferred to new cages and deprived of chow. Mice were fasted for 2 hours prior to receiving 250 µl of Moviprep, a polyethylene glycol-based laxative, at 30-minute intervals for a total of 5 administrations. Mice were then fasted for 6 additional hours before oral administration of 200 µl of fecal solution (fresh fecal pellets homogenized in sterile PBS 1× at 70–120 mg/ml and passed through a 70-µm filter) prepared using fecal pellets from 13 to 20 week-old WT or $sPLA_2$-IIA^{TGN} mice. The administration of fresh fecal solution was repeated on the two following days (9). Arthritis was induced two weeks later, and its severity was monitored daily for 9 days before mice were euthanized. The investigator assessing arthritis severity was blinded as to which mice were transplanted with which flora. Feces were collected throughout the experiment for microbiota composition assessment.

Intestinal permeability assessment

Arthritis was induced in 12–16-week-old male mice and severity of the disease was monitored daily. The mice acting as negative controls were injected with PBS 1× instead of arthritogenic K/B×N serum. Nine days following the first injection, mice were fasted for 4 hours and administered 600 mg/kg 4 kDa FITC-Dextran (FD4) via oral gavage. Blood was collected 4 hours later by cardiac puncture and serum was prepared. Serum fluorescence was measured in a plate reader at 485 nm excitation and 528 nm emission and FD4 concentration was determined using a serial dilution of FD4.

Whole-genome shotgun sequencing

Murine stool samples were collected upon defecation and were immediately frozen on dry ice. DNA was extracted using the DNeasy PowerSoil Kit (Qiagen), then quantified using QuantiFluor dye (Promega). Sequencing libraries were prepared using the Nextera Sample Preparation Kit (Illumina,

San Diego, CA, USA) with 50 ng of purified DNA. Dual indices were added during library preparation. Libraries were validated using Agilent Bioanalyzer 2100 high sensitivity DNA chips (Agilent Technologies, Santa Clara, CA, USA) and were re-quantified with QuBit (ThermoFisher Scientific). Libraries were diluted to a 2 nM concentration prior to pooling. Libraries were multiplexed and the pool was sequenced in replicate in two HiSeq lanes. Sequencing was performed on the HiSeq 2500 sequencer (Illumina) using v4 chemistry and 125 bp paired-end reads. A total of 16 metagenomes were sequenced with an average of 7 Gb per sample.

16S rRNA gene sequencing

Fecal DNA was extracted from frozen stool samples using the DNeasy PowerSoil Kit (Qiagen) and quantified using a BioDrop µLite (Isogen Life Science). To prepare 16S amplicon libraries, 15ng of DNA was used for an initial PCR amplification step of 25 cycles with specific bacterial 16S rRNA (V3-341F and V4-785R) hypervariable region oligos using Q5 High-Fidelity DNA polymerase (New England Biolabs, Whitby, Ontario, Canada). After purification with AxyPrep Mag PCR Clean-up kit (Axygen, Big Flats, NY, USA), a second PCR amplification step of 8 cycles was performed to incorporate specific index adaptors for multiplexing. This step was followed by a purification step using AxyPrep Mag PCR Clean-up kit (Axygen, Big Flats, NY, USA). The quality of the final libraries was examined with a DNA screentape D1000 on a TapeStation 2200 (Agilent Technologies, Santa Clara, CA, USA) and the quantification was performed with Quant-it HS (ThermoFisher Scientific, Canada) on a Victor3 plate reader fluorometer (PerkinElmer, Waltham, MA, USA). 16S libraries with unique indices were subsequently pooled in an equimolar ratio (75 samples/pool) and the pool was sequenced on an Illumina Miseq V3 system for 300 bp paired-end sequencing at the Next-Generation Sequencing Platform, Genomics Center, CHU de Québec-Université Laval Research Center, Québec City, Canada.

First PCR, stagger oligos with equimolar amounts between 0N, 1N, 2N and 3N

V3-341F forward primer: 5′-TCGTCGGCAGCGTCAGATGTGTATAAGAGACAG-3′ (0N–3N) 5′-CCTACGGGNGGCWGCAG-3′

V4-785R reverse primer: 5′-GTCTCGTGGGCTCGGAGATGTGTATAAGAGACAG-3′ (0N–3N) 5′-GACTACHVGGGTATCTAATCC-3′

Bioinformatic analysis

Shotgun sequencing analysis

The raw sequence data was subjected to quality control using FASTQC v0.11.2 (10). Quality filtration of fastq reads and adaptor removal was carried out using Trimmomatic v0.32 (11) with the following options: ILLUMINACLIP:2:30:1, TRAILING:30 and MINLEN:36. The reads from all samples were assembled using RayMeta v2.3.1 (12) with a k-mer size of 31 nucleotides. Quantification of the contigs was performed using kallisto v0.43.0. (13) Blastn v2.4.0 against nt was used to assign taxonomy to the contigs. Abundance counts were compiled using a custom R script and the PCA were produced using the FactoMineR v1.35 (14) package in R v3.2.2 (15).

16S sequencing analysis

The entire pipeline is accessible in supplemental data (**Supplemental Material 1**). An Amplicon Sequence Variant (ASV) methodology was used to analyze an average of 212 000 16S sequences using the DADA2 pipeline (16). Briefly, 16S amplicons were trimmed for their adapters using cutadapt (17) with the following options: -m 270 -M 330 --discard-untrimmed. The remaining reads were processed using DADA2 commands (16). The DADA2 pipeline allowed for the filtration and trimming of the data based on their quality profile, in addition to the removal of chimera using an error model based on the sequenced data. A count table of the retrieved ASVs was achieved using the makeSequenceTable function. A summary table of the counts at each step is accessible in supplemental data (**Supplemental Table 6**).

The ASVs were then analyzed using Blastn v2.4.0 against RefSeq nt for taxonomy annotation. Hits with a p-value > 10^{-5} were excluded from the analysis. ASV counts were normalized using the centered log ratio transformation (CLR) of the R package mixomics, and the beta diversity was calculated using the Bray-Curtis dissimilarity distance. Differential analysis was performed using the ALDEx package (18), allowing for the comparison of groups considered “prone to inflammation” and “less susceptible to inflammation” (**Figure 5**, Welch’s t test, p-value corrected by Benjamini-Hochberg FDR procedure).

Intestinal lipid profiling

To profile different lipids, frozen colon tissue samples (−196°C, ~10 mg) were crushed with a disposable tissue grinder, mixed with 500 µl PBS, then immediately denatured with 500 µl methanol containing 0.1% acetic acid and 5 ng of deuterated standards. The denatured samples were agitated at room temperature for 90 minutes then centrifuged (20,000 × *g*, 10 minutes). Lipids were extracted from the harvested liquid fractions by solid phase extraction using StrataX columns (60 mg, Phenomenex) as previously described (19). The resulting extracts were evaporated under a stream of nitrogen then suspended in 50 µl of a water:acetonitrile solution (50:50) containing 0.05% formic acid. Samples (40 µl) were injected onto a high-performance liquid chromatography (HPLC) column (Kinetex C8, 150 × 2.1 mm, 2.6 µm, Phenomenex) and eluted at a flow rate of 400 µl/min using a discontinuous gradient of solvent A (LC–MS grade water containing 0.05% formic acid) and solvent B (LC–MS grade acetonitrile containing 0.05% formic acid). Gradient was as follows: 10–25% B from 0 to 5 min, 25–35% B from 5 to 10 minutes; 35–75% B from 10 to 20 minutes, 75–95% from 20 to 20.1 minutes; 95% B from 20.1 to 25 minutes; 95–10% B from 25 to 25.1 minutes; 10% B from 25.1 to 28 minutes. The LC system was interfaced with the electrospray source of a Shimadzu 8050 triple quadrupole mass spectrometer and mass spectrometric analysis was performed using multiple reaction monitoring using the Shimadzu lipid mediator package (version 2).

Fecal lipidomic analysis

Fecal untargeted lipidomics was performed as previously described (20, 21). Murine fecal pellets were collected immediately after defecation, frozen on dry ice and stored at −80°C until they were processed for lipidomic analysis. Collected mice feces were homogenized using a multi-bead shocker (YASUI KIKAI, Osaka, Japan) for 15 sec at 2500 rpm. To the homogenized sample, 100 µL methanol per 10 mg-feces was added and was incubated overnight at -30°C. Whole lipids were extracted by single-phase extraction. Fecal suspension in methanol (200 µL) was mixed with 5 µL of EquiSPLASH (Avanti Polar Lipids, Alabama, USA), 10 µM of triple deuterium-labeled hexadecanoic acid and octadecanoic acid, 100 µL of chloroform and 20 µL of water, and the samples were centrifuged at 2000×*g* for 10 min to collect the supernatants. Untargeted lipidomics was performed by using an ACQUITY UPLC system (Waters, Milford, MA) coupled with a QTOF-MS (TripleTOF 6600; Sciex, Framingham, MA) as described previously (20). MS-DIAL bootstrap version 4.00 was used for data analyses (21). The following parameters were set: (data collection) MS1 tolerance, 0.01 Da; MS2 tolerance, 0.025 Da; (peak detection) minimum peak height, 500 amplitude; (Identification) retention time tolerance, 1.25 min; (alignment) retention time tolerance 0.05 min: MS1 tolerance, 0.015 Da, Default values were used for other parameters. Lipid quantification followed the definition of the Lipidomics Standards Initiative (LSI) (22): Level 2 quantification was achieved when lipid molecules are quantified by internal standards of the same lipid subclass, Level 3 quantification was achieved when lipid molecules were quantified by similar or representative lipid class internal standards.

Machine learning

Machine learning was used to identify a fecal lipid signature able to discriminate between mice expressing or not sPLA$_2$-IIA. We used BioDiscML (23), a machine learning sequential minimal optimization algorithm, to train a support vector classifier to identify a specific lipidic signature able to

discriminate between both groups using samples from the SPF and Elite facilities. Fecal lipid concentration data from middle-aged sPLA$_2$-IIA^{TGN} and WT mice housed in the SPF and Elite animal facilities quantified using lipidomic analysis was randomly split in two: 2/3 of the samples were used for training and the rest was used to test the predicted signature. Spearman and Pearson correlation >0.99 or similar information gain was used to create an extended signature. PCA and heatmap clusters were produced using the FactoMineR v1.35 package (14) and R v3.2.2 (15).

Supplemental references

1. Uozumi H et al. Role of cytosolic phospholipase A2 in allergic response and parturition. *Nature* 1997;390(6660):618–622.

2. Grass DS et al. Expression of human group II PLA2 in transgenic mice results in epidermal hyperplasia in the absence of inflammatory infiltrate.. *J. Clin. Invest.* 1996;97(10):2233–2241.

3. Nevalainen TJ et al. Expression of Human Group II Phospholipase A 2 in Transgenic Mice. *Histochemistry* 1997;45(8):1109–1119.

4. Duchez A-C et al. Respective contribution of cytosolic phospholipase A2α and secreted phospholipase A2 IIA to inflammation and eicosanoid production in arthritis. *Prostaglandins Other Lipid Mediat.* 2019;143:106340.

5. Duchez A-C et al. Platelet microparticles are internalized in neutrophils via the concerted activity of 12-lipoxygenase and secreted phospholipase A2 -IIA. *Proc. Natl. Acad. Sci.* 2015;112(27):E3564–E3573.

6. Ghomashchi F et al. Preparation of the Full Set of Recombinant Mouse- and Human-Secreted Phospholipases A2. In: *Methods in Enzymology*. 2017:35–69

7. Reikvam DH et al. Depletion of murine intestinal microbiota: Effects on gut mucosa and epithelial gene expression. *PLoS One* 2011;6(3):1–13.

8. Monach PA et al. The K/BxN arthritis model. *Curr. Protoc. Immunol.* 2008;(SUPPL. 81):1–12.

9. Le Roy T et al. Comparative evaluation of microbiota engraftment following fecal microbiota transfer in mice models: Age, kinetic and microbial status matter. *Front. Microbiol.* 2019;10(JAN):1–16.

10. Babraham Bioinformatics - FastQC A Quality Control tool for High Throughput Sequence

Data2019;http://www.bioinformatics.babraham.ac.uk/projects/fastqc/. cited December 3, 2019

11. Bolger AM et al. Trimmomatic: A flexible trimmer for Illumina sequence data. *Bioinformatics* 2014;30(15):2114–2120.

12. Boisvert S et al. Ray Meta: Scalable de novo metagenome assembly and profiling. *Genome Biol.* 2012;13(12):R122.

13. Bray NL et al. Near-optimal probabilistic RNA-seq quantification. *Nat. Biotechnol.* 2016;34(5):525–527.

14. Lê S et al. FactoMineR: An R package for multivariate analysis. *J. Stat. Softw.* 2008;25(1):1–18.

15. Core Team R. R: A Language and Environment for Statistical Computing. *R Found. Stat. Comput.* 2018;https://www.R-project.org/.

16. Callahan BJ et al. DADA2: High-resolution sample inference from Illumina amplicon data. *Nat. Methods* 2016;13(7):581–583.

17. Martin M. Cutadapt removes adapter sequences from high-throughput sequencing reads. *EMBnet.journal* 2011;17(1):10.

18. Fernandes AD et al. Unifying the analysis of high-throughput sequencing datasets: Characterizing RNA-seq, 16S rRNA gene sequencing and selective growth experiments by compositional data analysis. *Microbiome* 2014;2(1):15.

19. Everard A et al. Intestinal epithelial N-acylphosphatidylethanolamine phospholipase D links dietary fat to metabolic adaptations in obesity and steatosis. *Nat. Commun.* 2019;10(1). doi:10.1038/s41467-018-08051-7

20. Yasuda S et al. Elucidation of Gut Microbiota-Associated Lipids Using LC-MS/MS and 16S rRNA

Sequence Analyses. *iScience* 2020;23(12):101841.

21. Tsugawa H et al. A lipidome atlas in MS-DIAL 4. *Nat. Biotechnol.* 2020;38(10):1159–1163.

22. Liebisch G et al. Lipidomics needs more standardization. *Nat. Metab.* 2019;1(8):745–747.

23. Leclercq M et al. Large-scale automatic feature selection for biomarker discovery in high-dimensional omics data. *Front. Genet.* 2019;10(MAY):452.

24. Karlsson J et al. Regional variations in Paneth cell antimicrobial peptide expression along the mouse intestinal tract. *BMC Immunol.* 2008;9:37.

25. Castillo PA et al. An Experimental Approach to Rigorously Assess Paneth Cell α-Defensin (Defa) mRNA Expression in C57BL/6 Mice. *Sci. Rep.* 2019;9(1):1–14.

26. Yu S et al. The mRNA and Protein Levels of Tubulin and β-Actin Are Greatly Reduced in the Proximal Duodenum of Mice Relative to the Rest of the Small Intestines. *Dig. Dis. Sci.* 2015;60(9):2670–2676.

Supplemental figures

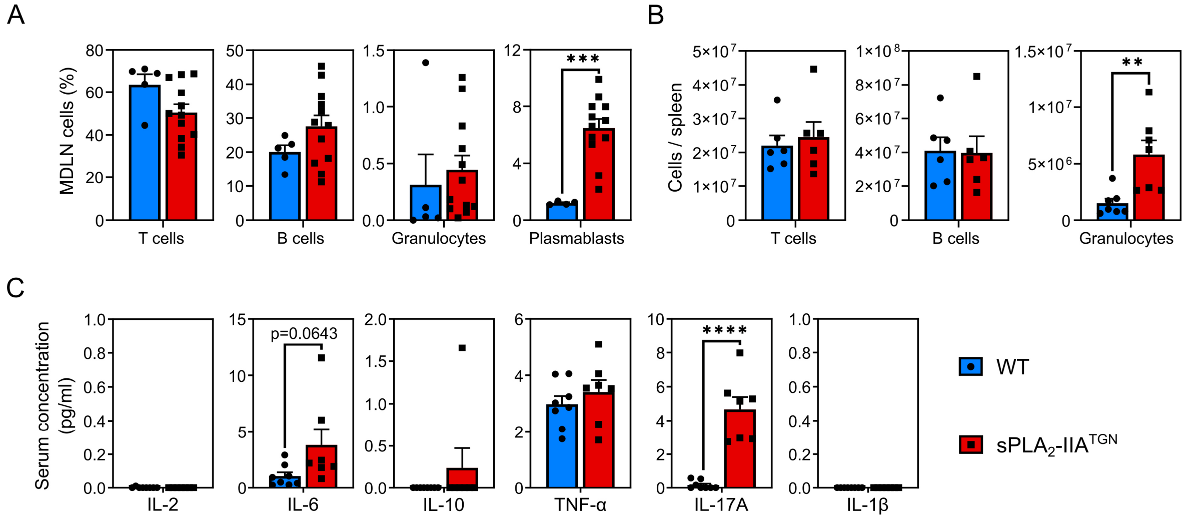

Supplemental Figure 1: Spontaneous immune phenotype in sPLA$_2$-IIA^{TGN} mice – Supplemental data. sPLA$_2$-IIA^{TGN} and WT mice were housed in an SPF animal facility for 8 months before assessment of the immune phenotype. (**A**) Flow cytometric analysis with markers targeting T-cells (CD3+ B220-), B-cells (B220+ CD3-), granulocytes (Gr1+) and plasmablasts (CD19+ CD138+). Cells were isolated from (**A**) MDLNs (n = 5–12) and (**B**) the spleen (n = 6–7) of WT and sPLA$_2$-IIA^{TGN} mice. Cell proportion is displayed for MDLNs and the cell count is shown for the spleen. (**C**) Cytokine concentration in the serum of 8-month-old WT and sPLA$_2$-IIA^{TGN} mice housed in the SPF animal facility (n = 7–8). Data are presented as mean +/– standard error of the mean (SEM). Statistical analysis: unpaired t test. **P < 0.01, ***P < 0.001, ****P < 0.0001.

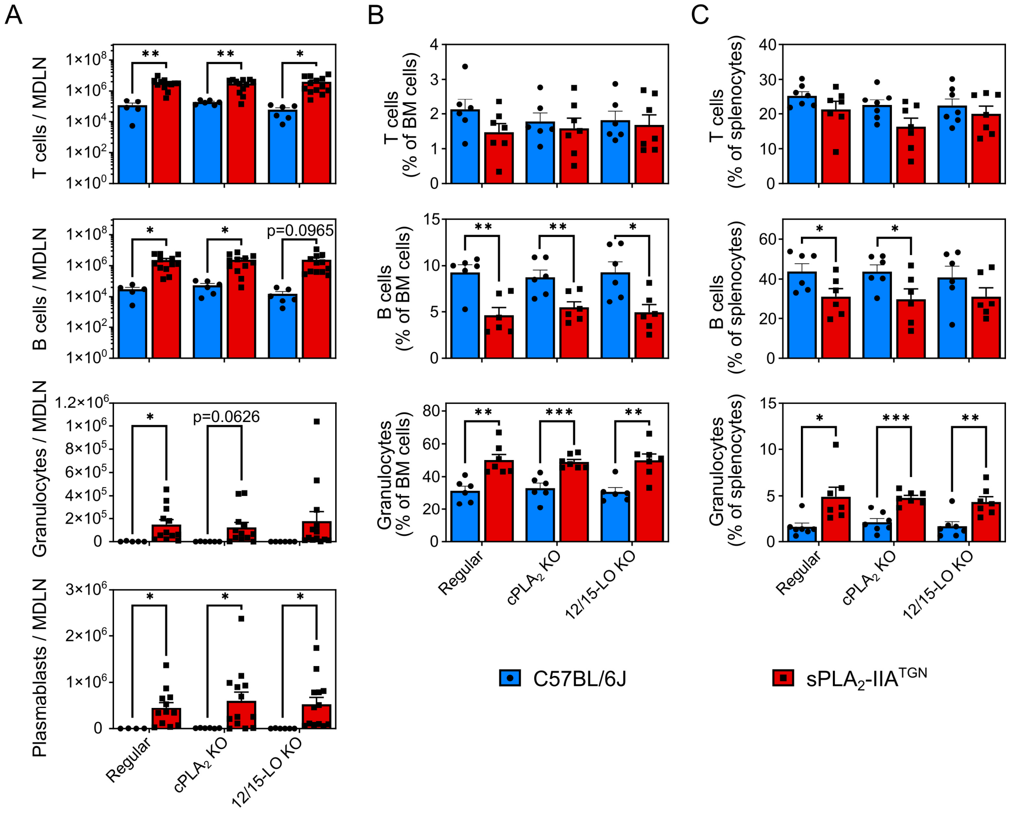

Supplemental Figure 2: cPLA2-α and 12/15-LO do not contribute to the sPLA2-IIA-mediated immune phenotype. sPLA2-IIA^{TGN} and C57BL/6J mice either expressing or deficient in cytosolic phospholipase A2-α and 12- and 15-lipoxygenase were housed in the SPF animal facility for 8 months before the severity of the immune phenotype was assessed. (**A**) Quantification of T-cells, B-cells, granulocytes and plasmablasts in MDLNs by flow cytometry. The proportions of T-cells, B-cells and granulocytes in (**B**) the bone marrow and (**C**) spleen were also assessed. Data are presented as mean +/– SEM. Statistical analysis: unpaired t test. *P < 0.05, **P < 0.01, ***P < 0.001.

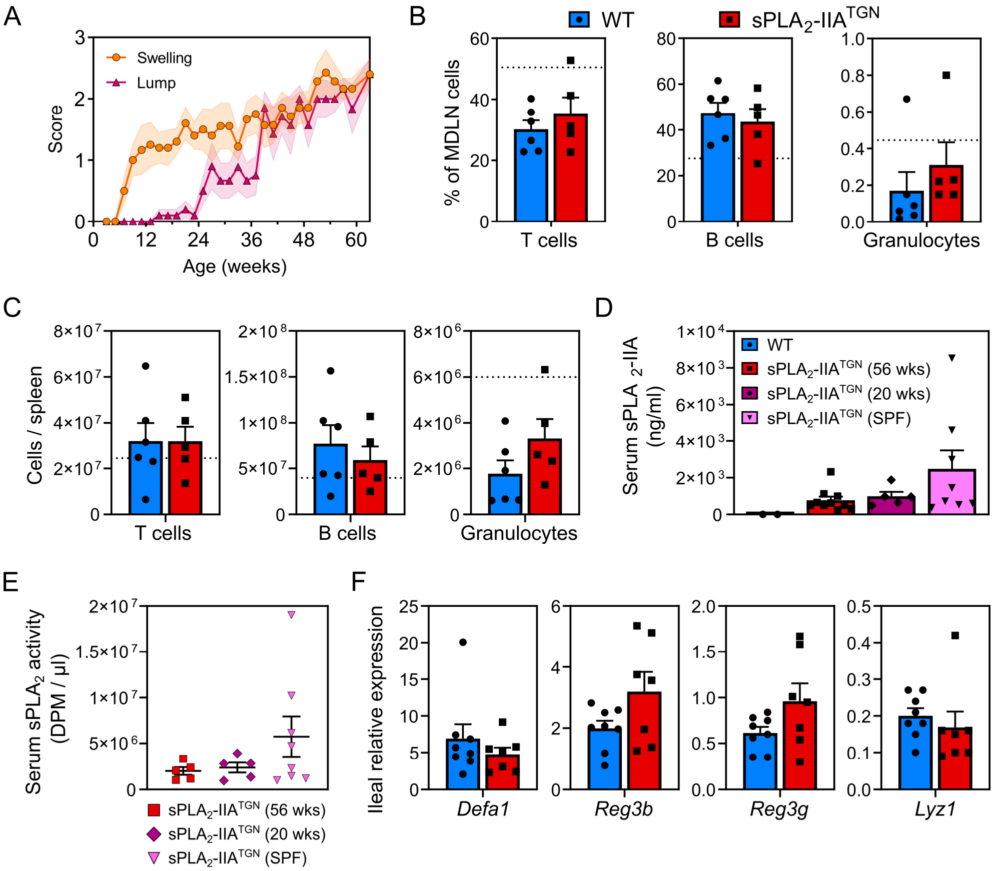

Supplemental Figure 3: sPLA2-IIA-mediated immune disorder in the Elite environment. Mice were housed in an Elite SPF+ animal facility for up to 63 weeks. (**A**) Bi-weekly scores were attributed to mice according to the swelling and the presence of lumps in the neck (n = 5–10). The scores ranged from 0 (no swelling or no perceivable lumps) to 3 (marked swelling that appeared uncomfortable for the mouse or solid lumps with diameter greater than 1 cm). (**B**) Proportion of T-cells, B-cells and granulocytes compared to total cells measured in MDLNs (n = 5–6) and (**C**) counts for those cell types in spleens from WT and sPLA2-IIA^{TGN} mice (n = 5–6). Dotted line represents the mean for sPLA2-IIA^{TGN} mice housed in the SPF animal facility. (**D**) sPLA2-IIA concentration measured in the serum of WT mice and 20- and 56-week-old sPLA2-IIA^{TGN} mice housed in the Elite facility in addition to 8-month-old sPLA2-IIA^{TGN} mice housed in the SPF animal facility (n = 2–10). (**E**) Enzymatic activity of serum samples on autoclaved *E. coli* membranes radiolabeled with [^{3}H]oleic acid, measured in disintegrations per minutes (DPM) from membrane lysate supernatant. (**F**) Relative expression of genes coding for antimicrobial peptides α-Defensin (*Defa1*), RegIIIβ (*Reg3b*), RegIIIγ (*Reg3g*) and lysozyme C (*Lyz1*) compared to the average expression of housekeeping genes β-actin, *Hprt* and 18S rRNA in the ileum of WT and sPLA2-IIA^{TGN} mice aged up to 63 weeks (n = 7–8). Data are presented as mean +/– SEM. Statistical analysis: unpaired t test.

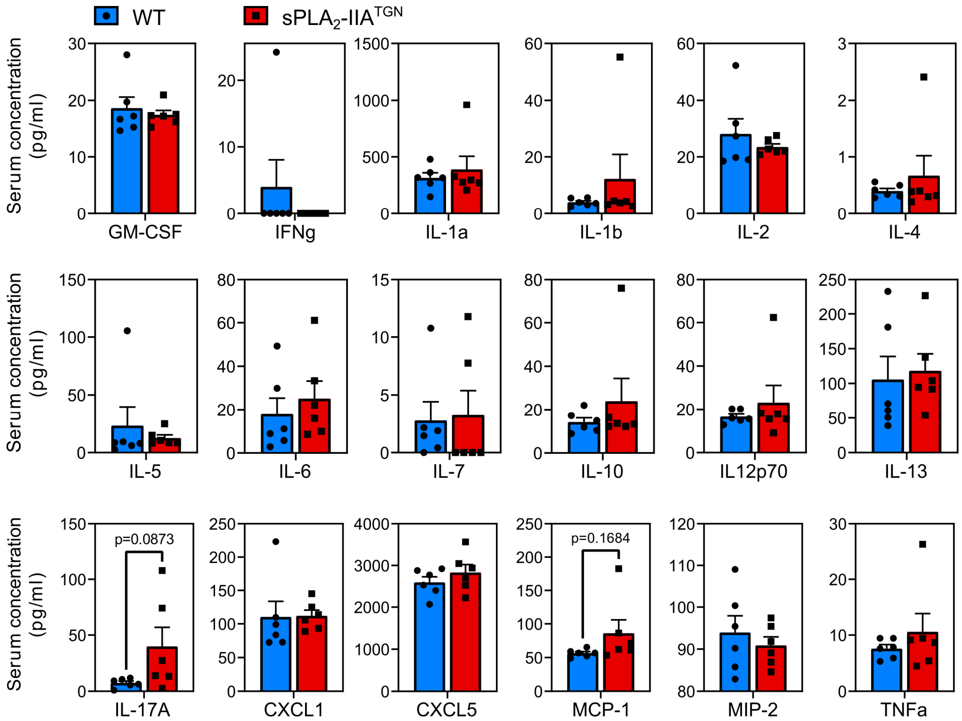

Supplemental Figure 4: T cell cytokine profiling in Elite sPLA$_2$-IIA^{TGN} mice. Cytokines were quantified in the serum of WT and sPLA$_2$-IIA^{TGN} mice housed in the Elite animal facility for ~8 months (n = 6) using an 18 plex T cell discovery array (Eve Technologies). Data are presented as mean +/− SEM. Statistical analysis: unpaired t test.

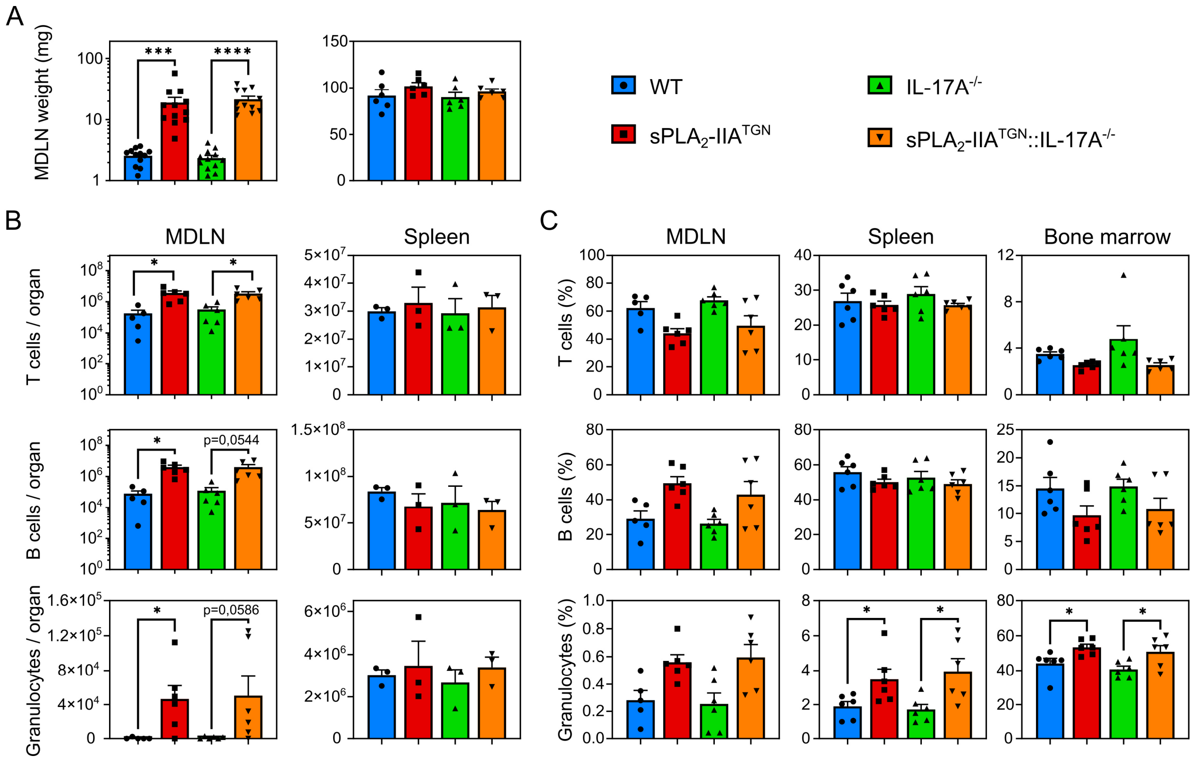

Supplemental Figure 5: IL-17A is not involved in the immune phenotype. (**A**) Weight of MDLNs (n = 12) and spleen (n = 6) of 7–8-month-old WT and sPLA$_2$-IIA^{TGN} mice housed in the Elite facility expressing or not IL-17A. (**B**) Quantification of T-cells, B-cells and granulocytes in MDLNs (n = 6) and spleen (n = 3) of all 4 mouse groups. (**C**) Cell proportions were also evaluated in MDLNs (n = 6), spleen (n = 6) and bone marrow (n = 6) of these groups. Data are presented as mean +/− SEM. Statistical analysis: unpaired t test. *P < 0.05, ***P < 0.001, ****P < 0.0001.

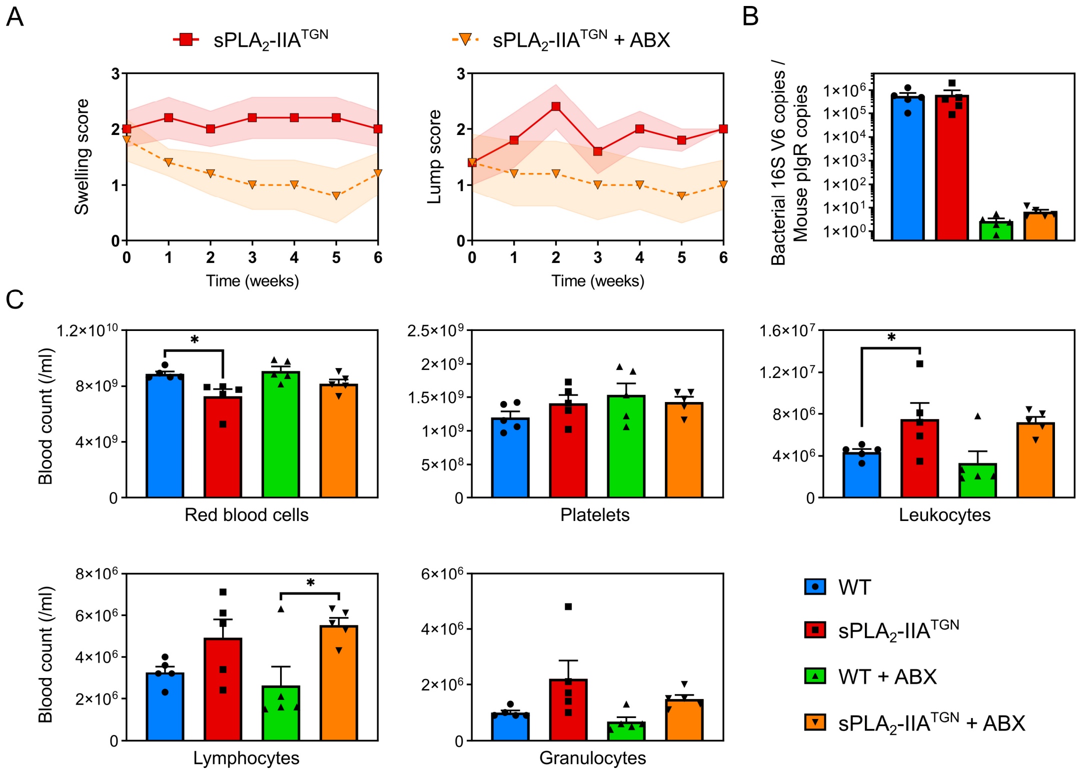

Supplemental Figure 6: Depletion of the microbiota in WT and sPLA2-IIA^{TGN} mice. 1-year-old WT and sPLA2-IIA^{TGN} mice housed in an Elite SPF+ animal facility received ampicillin in their drinking water and were administered neomycin, metronidazole and vancomycin via oral gavage twice daily for 6 weeks to achieve near-complete depletion of their intestinal flora. (**A**) Swelling and lump scores attributed to sPLA2-IIA^{TGN} mice treated or not with broad spectrum antibiotics (n = 5). (**B**) Ratio between the amount of bacterial DNA (number of copies of the variable region 6 of the 16S rRNA gene) and murine DNA (number of copies of mouse polymeric immunoglobulin receptor gene) in nucleic acids extracted from feces collected 6 weeks following the first antibiotic treatment (n = 5). (**C**) Blood composition of mice treated or not with antibiotics (n = 5). Data are presented as mean +/– SEM. Statistical analysis: unpaired t test. *P < 0.05.

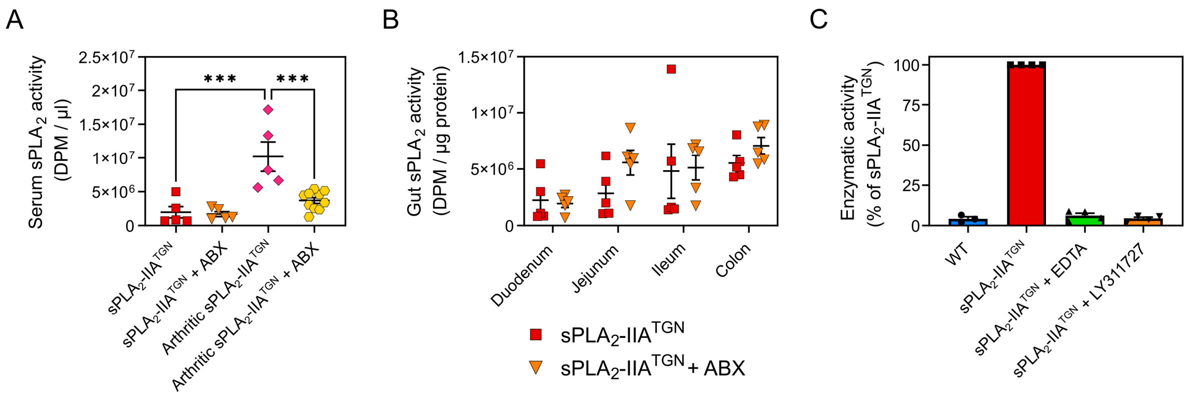

Supplemental Figure 7: Enzymatic activity of sPLA$_2$-IIA in sPLA$_2$-IIA^{TGN} mice. The sPLA$_2$ enzymatic activity was measured in (**A**) serum and (**B**) intestinal tissues from sPLA$_2$-IIA^{TGN} mice induced or not with arthritis and treated or not with broad spectrum antibiotics. Samples were incubated with autoclaved *E. coli* membranes radiolabeled with [^{3}H]oleic acid. Results are shown as disintegrations per minutes (DPM) from membrane lysate supernatant (n = 5–10). (**C**) The contribution of sPLA$_2$-IIA to the measured activity was assessed in intestinal samples from sPLA$_2$-IIA^{TGN} mice and compared to samples treated with an inhibitor of sPLA$_2$ (EDTA) and a specific inhibitor of sPLA$_2$-IIA (LY311727) (n = 3–4). Data are presented as mean +/− SEM. Statistical analysis: unpaired t test. ***P < 0.001.

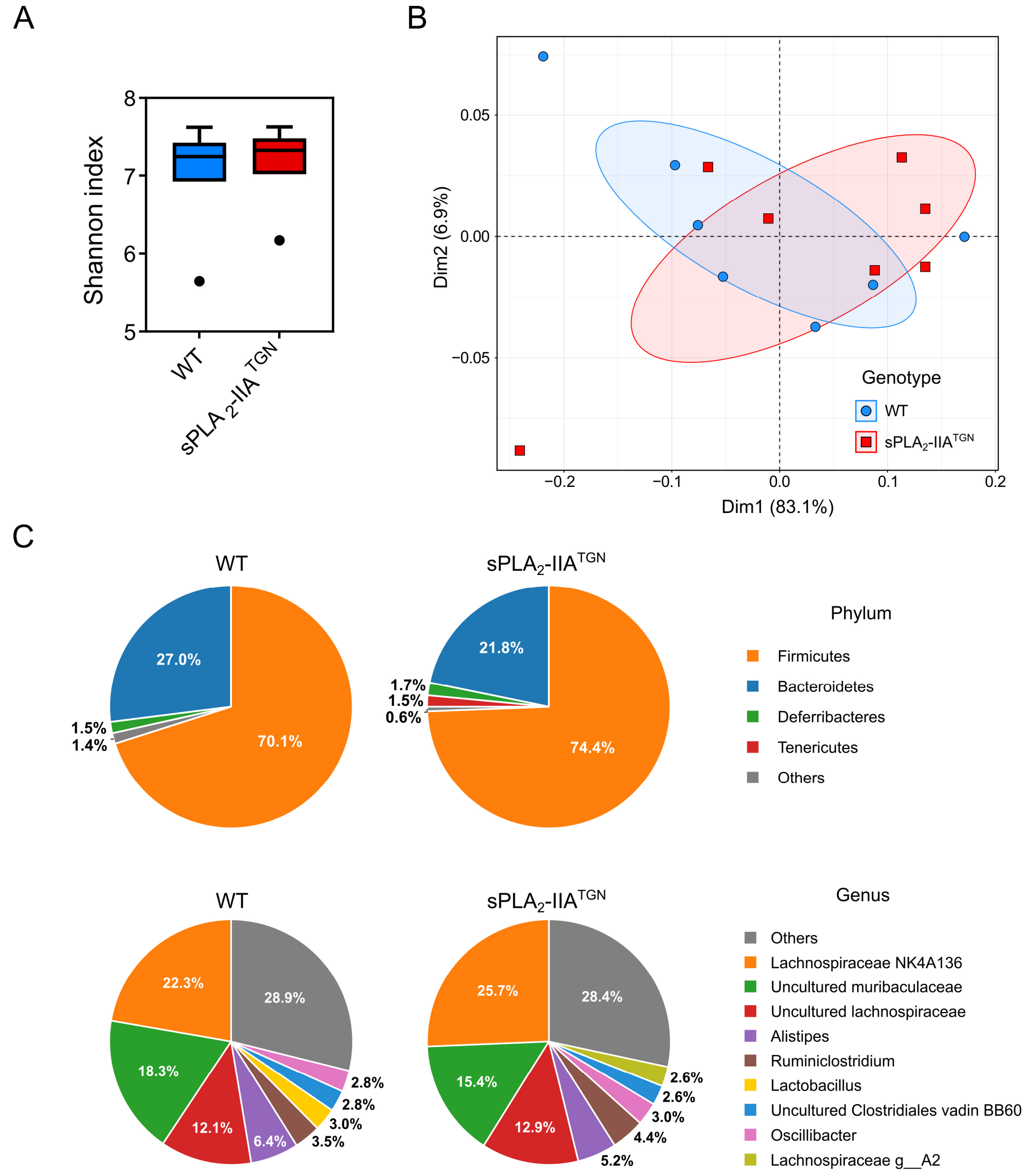

Supplemental Figure 8: The intestinal microbiota of sPLA$_2$-IIA^{TGN} mice is not altered in the Elite environment. The fecal microbiota of 14-month-old sPLA$_2$-IIA^{TGN} and WT mice housed in an Elite SPF+ animal facility was sequenced by 16S rRNA gene sequencing and the composition of this flora was identified. (**A**) Alpha diversity (Shannon index) of the microbiome of each mouse group. (**B**) Visualization of the data distribution using a principal component analysis with 99% confidence ellipses based on the relative abundance of quantified OTUs. (**C**) Relative abundance of identified bacterial phyla and genera in WT and sPLA$_2$-IIA^{TGN} mice (n = 7). Differential enrichment analysis revealed no alterations between the microbiome of each mouse group. (**A**) Data are presented as quartiles with whiskers extending to 1.5 inter-quartile range.

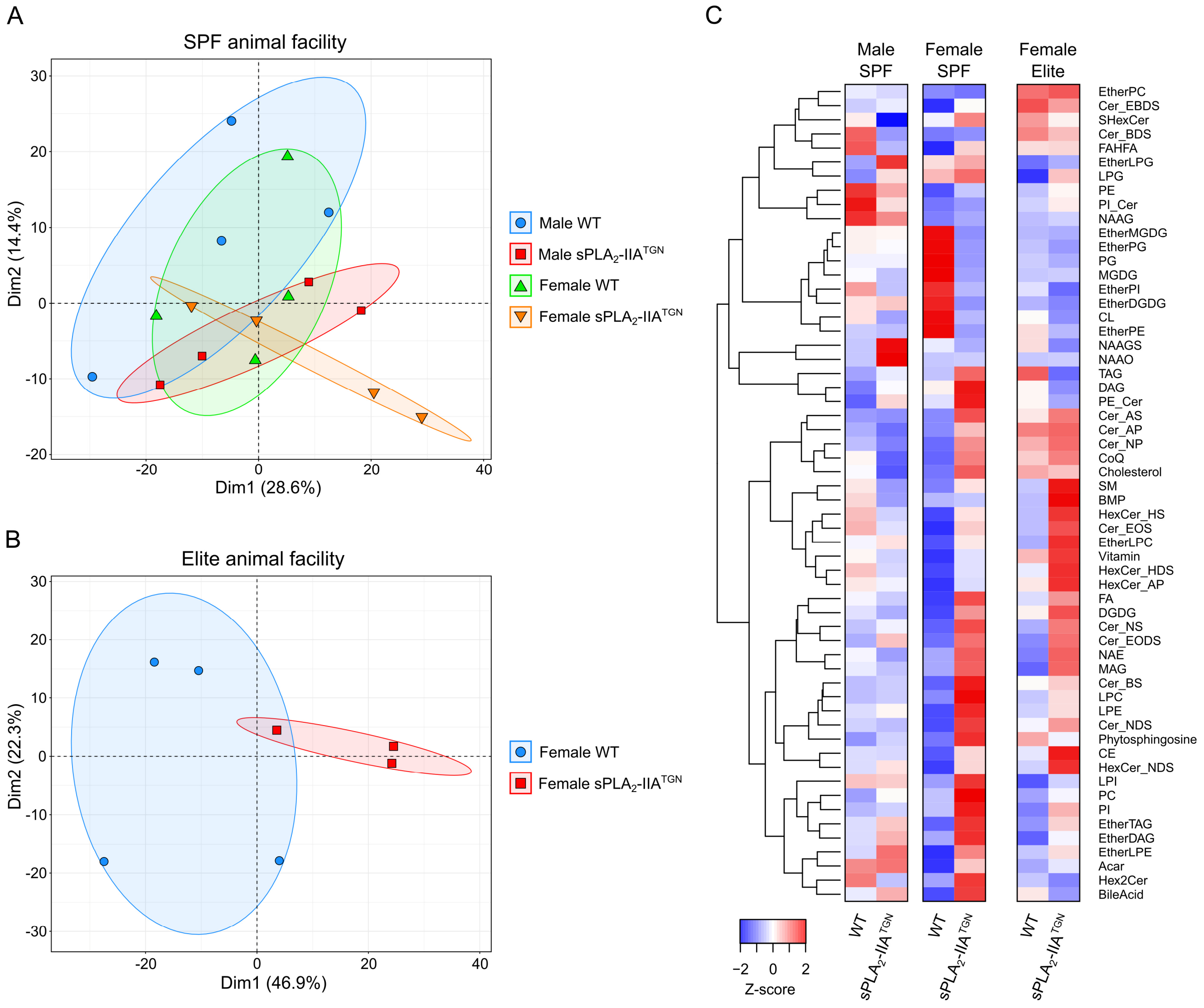

Supplemental Figure 9: Fecal lipid profile of SPF-housed mice and female Elite-housed mice. Untargeted lipidomics were used to characterize the fecal lipid profile of 8-month-old mice housed in the SPF animal facility and 14-month-old females housed in the Elite facility using high-performance liquid chromatography combined with mass spectrometry. The data distributions for (**A**) male and female SPF mice (n = 4) and (**B**) female Elite mice (n = 3–4) were visualized by principal component analysis (PCA) with 99 % confidence ellipses. (**C**) Heatmap generated using z-scores calculated from the concentration of measured lipid classes in each experimental group.

```
SMO classifier (Sequential Minimal Optimization algorithm for training a support vector
classifier)

Kernel used:
  Linear Kernel: K(x,y) = <x,y>

Classifier for classes: sPLA2-IIATGN, WT

BinarySMO

Machine linear: showing attribute weights, not support vectors.

        -0.4628 * (normalized) Cer-AS_d50:1+O;_Cer-AS_d18:1/32:0+O;_[M+CH3COO]-
 +      -0.4594 * (normalized) Cer-EODS_d50:3;_Cer-EODS_d32:1-O-18:2;_[M+CH3COO]-
 +      -0.3972 * (normalized) Cer-NDS_d52:1;_[M+CH3COO]-
 +      -0.2018 * (normalized) Cer-NS_d50:2;_Cer-NS_d18:1/32:1;_[M+CH3COO]-
 +      -0.266  * (normalized) DAG_34:0e;_DAG_16:0e/18:0;_[M+NH4]+
 +      -0.3311 * (normalized) DAG_36:0e;_DAG_16:0e/20:0;_[M+NH4]+
 +       1.1588 * (normalized) DAG_36:2e;_DAG_20:2e/16:0;_[M+NH4]+
 +      -0.3619 * (normalized) DAG_38:1e;_DAG_18:0e/20:1;_[M+NH4]+
 +      -0.5305 * (normalized) DAG_40:1e;_DAG_20:0e/20:1;_[M+NH4]+
 +      -0.4533 * (normalized) DAG_42:1e;_DAG_18:0e/24:1;_[M+NH4]+
 +      -0.9154 * (normalized) DAG_42:2e;_DAG_22:1e/20:1;_[M+NH4]+
 +      -0.4994 * (normalized) DAG_44:1e;_DAG_20:0e/24:1;_[M+NH4]+
 +      -0.7567 * (normalized) DAG_44:2e;_DAG_24:1e/20:1;_[M+NH4]+
 +      -0.3218 * (normalized) DAG_46:1e;_DAG_24:1e/22:0;_[M+NH4]+
 +      -0.7892 * (normalized) DAG_46:2e;_DAG_22:1e/24:1;_[M+NH4]+
 +      -0.2511 * (normalized) DAG_48:1e;_DAG_24:1e/24:0;_[M+NH4]+
 +      -0.48   * (normalized) DAG_48:2e;_DAG_24:1e/24:1;_[M+NH4]+
 +      -0.4268 * (normalized) DAG_50:2e;_DAG_24:1e/26:1;_[M+NH4]+
 +       0.1959 * (normalized) FA_22:1;_[M-H]-
 +       0.0921 * (normalized) FA_28:1;_[M-H]-
 +       0.0935 * (normalized) FA_30:1;_[M-H]-
 +      -0.2874 * (normalized) FA_30:2;_[M-H]-
 +       0.2178 * (normalized) FA_32:0;_[M-H]-
 +      -0.3522 * (normalized) FA_32:2;_[M-H]-
 +      -0.1997 * (normalized) FA_34:1;_[M-H]-
 +      -0.3369 * (normalized) FA_34:2;_[M-H]-
 +      -0.1607 * (normalized) FA_34:3;_[M-H]-
 +      -0.0519 * (normalized) FA_34:4;_[M-H]-
 +      -0.4623 * (normalized) FA_36:2;_[M-H]-
 +      -0.1453 * (normalized) FA_36:3;_[M-H]-
 +      -0.5838 * (normalized) PE-Cer_d33:1+O;_PE-Cer_d18:0/15:1+O;_[M-H]-
 +      -0.2496 * (normalized) TAG_54:1e;_TAG_18:0e-16:0-20:1;_[M+NH4]+
 +      -0.4224 * (normalized) TAG_56:1e;_TAG_18:0e-18:0-20:1;_[M+NH4]+
 +      -0.2409 * (normalized) TAG_58:1e;_TAG_18:0e-20:0-20:1;_[M+NH4]+
 +      -0.1508 * (normalized) TAG_58:3e;_TAG_20:0e-20:1-18:2;_[M+NH4]+
 +       1.1183

Number of kernel evaluations: 139 (75.442% cached)
```

Supplemental Figure 10: Fecal lipid signature distinguishing WT from sPLA$_2$-IIA^{TGN} mice. Report generated following identification of a signature able to discriminate between middle-aged WT and sPLA$_2$-IIA^{TGN} mice (n = 15–16) from both the SPF and Elite animal facilities using BioDiscML (23).

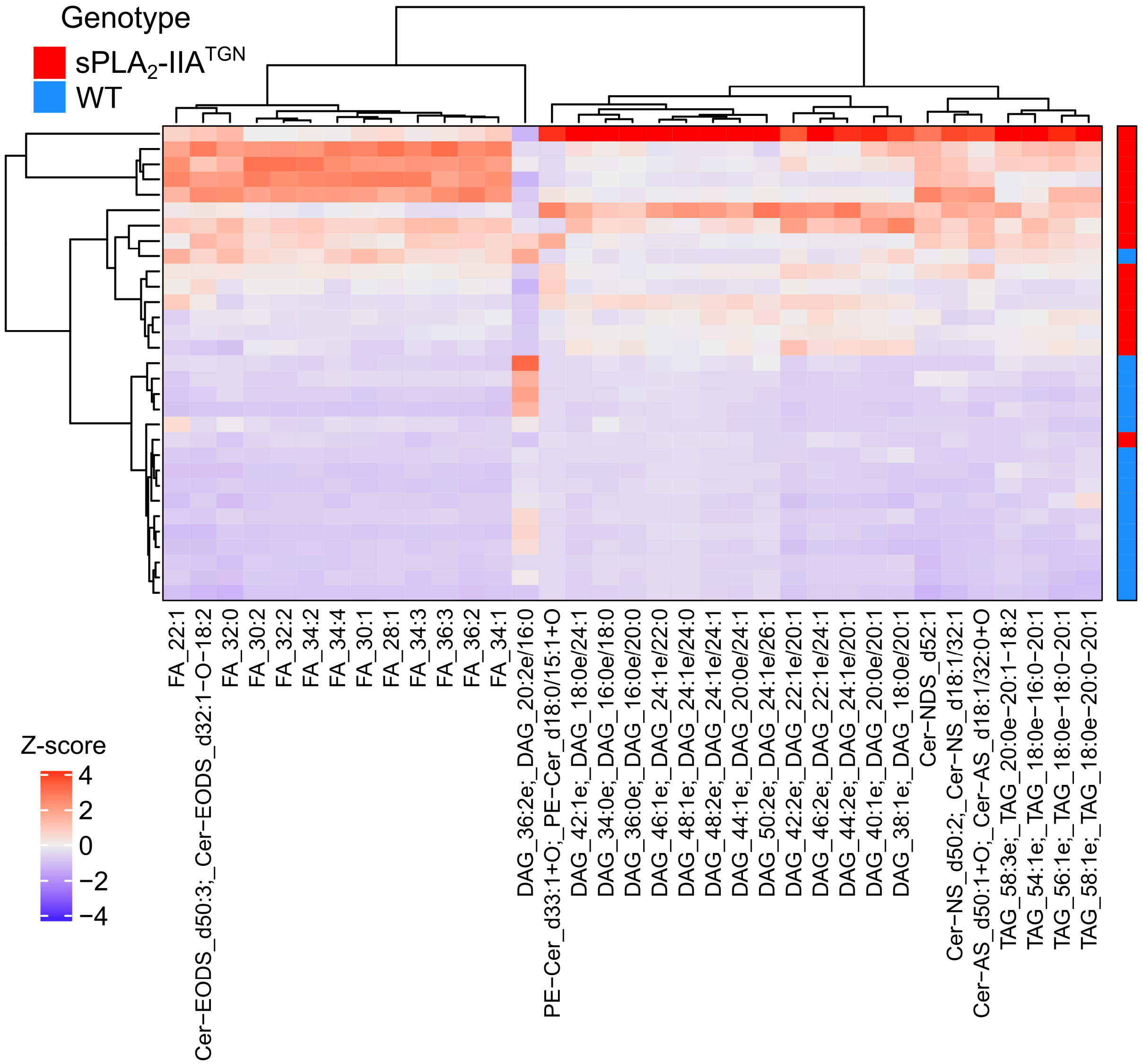

Supplemental Figure 11: Extended lipid signature able to discriminate between mice expressing or not sPLA$_2$-IIA. An extended fecal lipid signature able to distinguish WT and sPLA$_2$-IIA^{TGN} mice independently of their housing facility and sex was generated by machine learning, to which 27 addition metabolites were added using Spearman and Pearson correlation >0.99 and (n = 15–16 non-arthritic WT and sPLA$_2$-IIA^{TGN} mice housed in either the SPF or Elite animal facility for 8 or 14 months respectively). Identified metabolites are presented as a heatmap of the z-scores calculated from the concentration of the lipids.

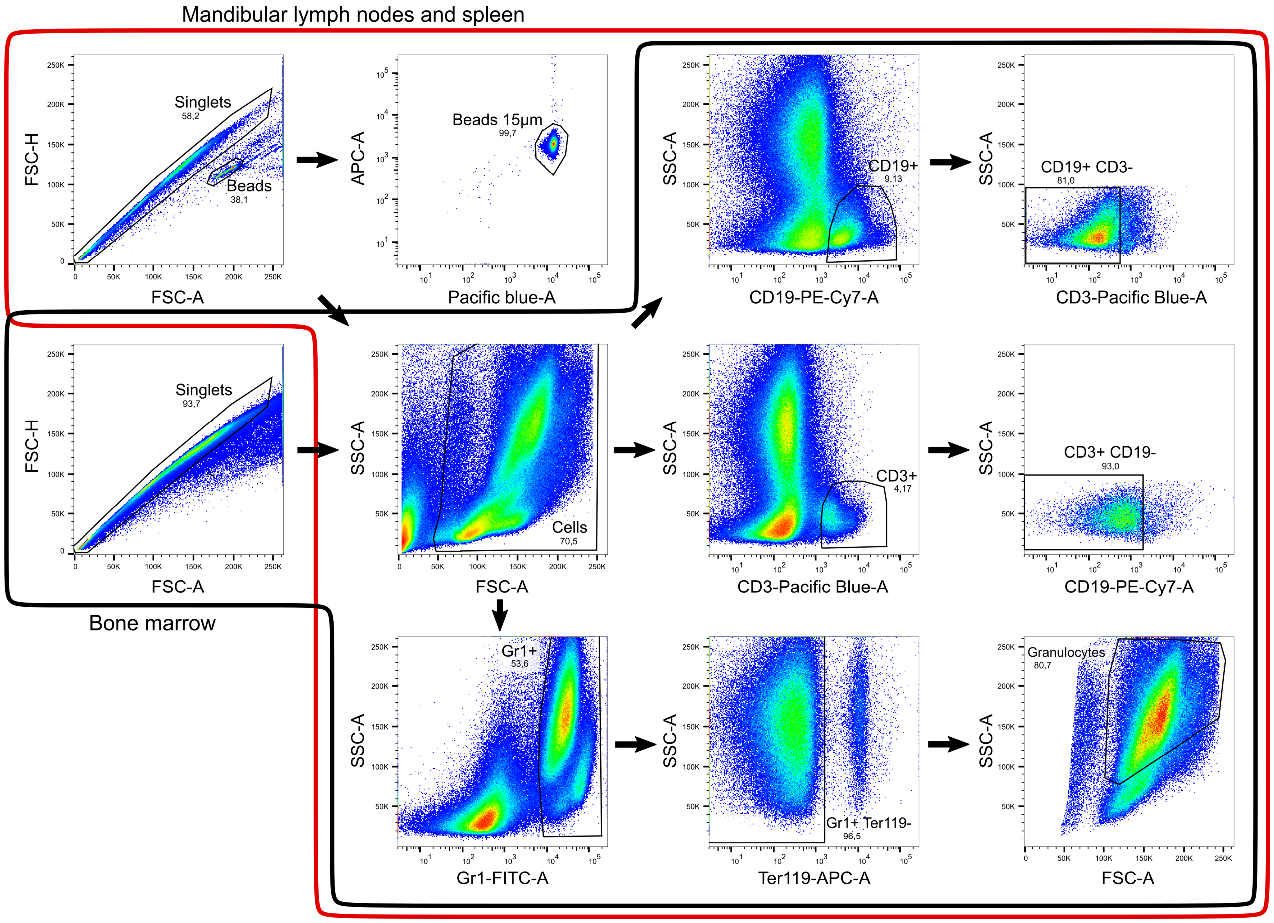

Supplemental Figure 12: Flow cytometry gating strategy. Gating strategy used to differentiate T cells (CD3+ CD19-), B cells (CD19+ CD3-) and granulocytes (Gr1+ Ter119-) in cells isolated from bone marrow (black frame) or mandibular lymph nodes and spleen (red frame) from mice.

16S rRNA V6 FOR

CGAAAGCGTGGGGAGCAAAC**AGGATTAGATACCCTGGTAGTCCACGCCGTAAACGAT**

GTCGACTTGGAGGTTGTGCCCTTGAGGCGTGGCTTCCGGAGCTAACGCGTTAAGTCGA

CCGCCTGGGGAGTACGGCCGCAAGGTTAAAACTCAAATGAATTGACGGGGGCCCGCA

CAAGCGGTGGAGCATGTGGTTTAATTCGATGCAACGCGAAGAACCTTACCTGGTCTTG

ACATCCACAGAACTTTCCAGAGATGGATTGGTGCCTTCGGGAAC TGTGAGACAGGTGC

16S rRNA V6 REV

TGCATGGCT GTCGTCAGCTCGTGTTGGTCGATAGTCAGGCAGCTTCTTTGCTGAC GAGT

GGCGGACGGGTGAGTAATGTCTGGGAAACTGCCTGATGGAGGGGGATAACTACTGGA

AACGGTAGCTAATACCGCATAACGTCGCAAGACCAAAGAGGGGGACCTTCGGGCCTCTT

GCCATCGGATGTGCCCAGATGGGATTAGCTAGTAGGTGGGGTAACGGCTCACCTAGGC

GACGATCCC TAGCTGGTCTGAGAGGATGACCAGCCACACTGGAACTGAGACACGGTCC

AGACTCCTACGGGAGGCAGCAGTGGGGAATATTGC ACAATGCTCCCATCAGAGCTG **TTT**

m-pIgR FOR

GCTCCTGGGCCTCCAAGTTCTGTTACTTACCCCTGCTTAGGGCCTATCCATGACTAGTGA

m-pIgR REV

CTCCTTGATTTGTGGCAGTCACGGGCT CAGGTGCCCCCTGAGTCTCC

Supplemental Figure 13: DNA sequence of the gBlock Gene Fragment used for the standard curve for the assessment of microbiota depletion. gBlock designed to contain the sequence of variable region 6 of the 16S rRNA gene (16S rRNA V6) of *Escherichia coli* (in blue) and a sequence from the mouse polymeric immunoglobulin receptor gene (m-pIgR, in green). Forward (FOR) and reverse (REV) primers are underlined and identified.

Supplemental tables

Supplemental Table 1: Bacterial exclusion list for the SPF and Elite SPF+ animal facilities at CHU de Québec-Université Laval. Microorganisms excluded from the animal facilities are marked with a "-", bacteria marked with a "+/–" may be present as they are tolerated depending on multiple parameters (room status, housing conditions, impact on current and future protocols), and tolerated bacteria are marked with a "+".

Virus	SPF	Elite SPF+
Ectromelia virus	-	-
Encéphalomyélite murine	-	-
Hantaan virus (hantavirus)	-	-
Mouse K virus	-	-
Lactic dehydrogenase elevating virus	-	-
Lymphocytic choriomeningitis virus	-	-
Mouse adenovirus	-	-
Mouse cytomegalovirus	-	-
Mouse hepatitis virus	-	-
Mouse minute virus	-	-
Mouse norovirus	-	-
Mouse parvovirus	-	-
Mouse thymic virus	-	-
Pneumonia virus of mice	-	-
Polyoma virus	-	-
Reovirus 3	-	-
Mouse Rotavirus	-	-
Sendai virus	-	-

Bacteria or fungi	SPF	Elite SPF+
Cilia Associated Respiratory bacillus	-	-
Citrobacter rodentium	-	-
Clostridium piliforme	-	-
Cornebacterium kutscheri	-	-
Mycoplasma pulmonis	-	-
Salmonella spp.	-	-
Streptobacillus moniliformis	-	-
Klebsiella pneumoniae	+/-	-
Klebsiella spp.	+/-	-
Pasteurella pneumotropica	+/-	-
Pneumocystis murina	+/-	-
Pseudomonas aeruginosas	+/-	-
Pseudomonas spp.	+/-	-
Bordetella bronchiseptica	+/-	-
Cornebacterium bovis	+	-
Streptococcus spp.	+	-
Staphylococcus aureus	+	+/-

Supplemental Table 5: qPCR and RT-qPCR primers used in this study

Target	Primers	Usage	Source
16S rRNA V5-6 region	F: 5′-AGGATTAGATACCCTGGTA-3′<br>R: 5′-CRRCACGAGCTGACGAC-3′	qPCR	(7)
Mouse polymeric immunoglobulin receptor (m-pIgR)	F: 5′-TTTGCTCCTGGGCCTCCAAGTT-3′<br>R: 5′-AGCCCGTGACTGCCACAAATCA-3′	qPCR	(7)
α-Defensin	F: 5′-TCAAGAGGCTGCAAAGGAAGAGAAC-3′<br>R: 5′-TGGTCTCCATGTTCAGCGACAGC-3′	RT-qPCR	(24)
RegIIIβ	F: 5′-CCTTAGACCGTGCTTTCTGTG-3′<br>R: 5′-GTCCATGATGCTCTTCAAGACA-3′	RT-qPCR	(7)
RegIIIγ	F: 5′-ACATCAACTGGGAGACGAATC-3′<br>R: 5′-TTTGGGATCTTGCTTGTGGCTA-3′	RT-qPCR	(7)
Lysozyme C type P	F: 5′-GCCAAGGTCTAACAATCGTTGTGAGTTG-3′<br>R: 5′-CAGTCAGCCAGCTTGACACCACG-3′	RT-qPCR	(24)
β-Actin	F: 5′-GCTGAGAGGGAAATCGTGCGTG-3′<br>R: 5′-CCAGGGAGGAAGAGGATGCGG-3′	RT-qPCR	(25)
18S rRNA	F: 5′-GCTTAATTTGACTCAACACGGGA-3′<br>R: 5′-AGCTATCAATCTGTCAATCCTGTC-3′	RT-qPCR	(26)
Hprt	F: 5′-TGATCAGTCAACGGGGGACA-3′<br>R: 5′-TTCGAGAGGTCCTTTTCACCA-3′	RT-qPCR	(7)
