# Supplementary material for: The interaction of secreted phospholipase A2-IIA with the microbiota alters its lipidome and promotes inflammation
Source: JCI Insight. 2022 Jan 25;7(2):e152638. doi: 10.1172/jci.insight.152638 (PMC8855825; doi:10.1172/jci.insight.152638)
Supplement: Supplemental data set 1 [file jciinsight-7-152638-s156.pdf]

CUTADAPT in bash
```
for sample in $(cat samples)
do
    echo "On sample: $sample"
    cutadapt -a ^cctacgggnggcwgcag...GGATTAGATACCCBDGTAGTC -A
^gactachvgggtatctaatcc...CTGCWGCCNCCCGTAGG -m 270  -M 330 --discard-
untrimmed -o ${sample}_R1_trimmed.fq.gz -p ${sample}_R2_trimmed.fq.gz
${sample}_L001_R1_001.fastq.gz ${sample}_L001_R2_001.fastq.gz  >>
cutadapt_primer_trimming_stats.txt 2>&1
done
```
DADA2 processing in R
```
library(dada2)
setwd("/is3/projects/AD_EB_mouse16S_2020/qiime2/rawdata")
samples <- scan("samples", what="character")
filtered_forward_reads <- paste0(samples, "_R1_filtered.fq.gz")
 filtered_reverse_reads <- paste0(samples, "_R2_filtered.fq.gz")
> filtered_out <- filterAndTrim(forward_reads, filtered_forward_reads,
+                 reverse_reads, filtered_reverse_reads, maxEE=c(2,2),
+                 rm.phix=TRUE, minLen=200, truncLen=c(250,200))

err_forward_reads <- learnErrors(filtered_forward_reads,
multithread=TRUE)
err_reverse_reads <- learnErrors(filtered_reverse_reads,
multithread=TRUE)

derep_forward <- derepFastq(filtered_forward_reads, verbose=TRUE)
names(derep_forward) <- samples # the sample names in these objects
are initially the file names of the samples, this sets them to the
sample names for the rest of the workflow
derep_reverse <- derepFastq(filtered_reverse_reads, verbose=TRUE)
names(derep_reverse) <- samples

dada_forward <- dada(derep_forward, err=err_forward_reads,
pool="pseudo", multithread=TRUE)
dada_reverse <- dada(derep_reverse, err=err_reverse_reads,
pool="pseudo", multithread=TRUE)

merged_amplicons <- mergePairs(dada_forward, derep_forward,
dada_reverse,
                    derep_reverse, trimOverhang=TRUE, minOverlap=20)

seqtab <- makeSequenceTable(merged_amplicons)
seqtab.nochim <- removeBimeraDenovo(seqtab, verbose=T)


sum(seqtab.nochim)/sum(seqtab)
```

getN <- function(x) sum(getUniques(x))
```
## making a little table
```
summary_tab <- data.frame(row.names=samples,
dada2_input=filtered_out[,1],
               filtered=filtered_out[,2], dada_f=sapply(dada_forward,
getN),
                dada_r=sapply(dada_reverse, getN),
merged=sapply(merged_amplicons, getN),
               nonchim=rowSums(seqtab.nochim),

final_perc_reads_retained=round(rowSums(seqtab.nochim)/
filtered_out[,1]*100, 1))

save(list = ls(), file = "env_entier.RData")

asv_seqs <- colnames(seqtab.nochim)
asv_headers <- vector(dim(seqtab.nochim)[2], mode="character")

for (i in 1:dim(seqtab.nochim)[2]) {
    asv_headers[i] <- paste(">ASV", i, sep="_")
  }
```
## count table:
```
asv_tab <- t(seqtab.nochim)
  row.names(asv_tab) <- sub(">", "", asv_headers)
  write.table(asv_tab, "ASVs_counts.tsv", sep="\t", quote=F,
col.names=NA)
```
## making and writing out a fasta of our final ASV seqs:
```
asv_fasta <- c(rbind(asv_headers, asv_seqs))
write(asv_fasta, "ASVs.fa")
```
#######################################
# Beta diversity, differential analysis: Generating figures
#######################################
```
library (vegan)
library(RColorBrewer)
library(ggplot2)
library(phyloseq)
library("tibble")
library("stringr")
library("ggrepel")
library("tidyverse")
library("ALDEx2")
library(ggh4x)

library("taxonomizr")
library(ggfortify)
library("mixOmics")
theme_set(theme_bw())
getPalette = colorRampPalette(brewer.pal(12, "Set3"))
```

## Metadata

```
metadata <- read_tsv("/Users/alban/Documents/souris_arhtrite/qiime2/metadata.txt")
colnames(metadata) = str_replace_all(colnames(metadata), " ", "_")
colnames(metadata) = str_replace_all(colnames(metadata), "Sample", "sample")
metadata$sample_name =str_replace_all(metadata$sample_name, "-", "_")
```

BLAST

```
blast = read_tsv("/Users/alban/Documents/souris_arhtrite/qiime2/ASV_annotation_blastout.txt", col_names = FALSE)
colnames(blast) = c("ASVs", "geneID", "percent", "cov_length", "mismatch" ,
"gapopen","qstart","qend","sstart","send","evalue","bitscore","query_length")
tax = read_tsv("/Users/alban/Documents/souris_arhtrite/qiime2/taxid_matched", col_names = FALSE) %>% dplyr::select(X2,X3)
colnames(tax) = c("geneID", "taxid")
names_dmp = read.names("/Users/alban/Documents/souris_arhtrite/shotgun_souris/names.dmp", onlyScientific = TRUE)
colnames(names_dmp) = c("taxid", "name_tax")
```

data loading

```
rawdata <- read.table("/Users/alban/Documents/souris_arhtrite/qiime2/summary_tab_qiime2.tsv", row.names = 1, sep = "\t" )
rownames(rawdata) = str_remove(rownames(rawdata), regex("_S.*$"))
rownames(rawdata) = str_replace_all(rownames(rawdata), "-", "_")
abun = read_tsv("/Users/alban/Documents/souris_arhtrite/qiime2/ASVs_counts.tsv") %>%
  column_to_rownames(var = "X1")

colnames(abun)  = str_replace_all(colnames(abun),regex("_S.*$"), "")
colnames(abun)  = str_replace_all(colnames(abun),"-", "_")

abun_filt =  abun[rowSums(abun > 0) / (ncol(abun)) > 0.5,]

```
abun_mind15 = dplyr::select(abun_filt, -contains("_D15"))

coldata = lapply(metadata, function(x) str_replace_all(x, " ", "_"))
%>% as.data.frame()
coldata = lapply(coldata, function(x) str_replace_all(x, "-", "_"))
%>% as.data.frame()
coldata = mutate(coldata, gt = paste(Group, Timepoint, "_"))
```
## CLR Normalization
```
abun_out.clr = logratio.transfo(as.matrix(t(abun_mind15)), logratio =
'CLR', offset = 1)
abun_out.clr_final = abun_out.clr + min(abun_out.clr) %>% abs()
class(abun_out.clr_final) <- "matrix"
d = as.matrix(abun_out.clr_final) %>% as.data.frame()
coldata = lapply(metadata, function(x) str_replace_all(x, " ", "_"))
%>% as.data.frame()
coldata = lapply(coldata, function(x) str_replace_all(x, "-", "_"))
%>% as.data.frame()
coldata = mutate(coldata, gt = paste(Group, Timepoint, "_"))
coldata_mind15 = filter(coldata, !str_detect(sample_name, "D15"))
```

Beta Diversity
```
beta = vegdist(as.matrix(abun_out.clr_final), method="bray")
betaDistMatrix <- as.matrix(beta) %>% as.data.frame()
colors <- colorRampPalette( rev(brewer.pal(9, "Greys")) )(255)
my_colour = list(
  Group = c(Donor_sPLA2_IIATGN = "#FFD700",Donor_WT = "#40E0D0"  ,
            `sPLA2_IIATGN__>_sPLA2_IIATGN` = "#FF0000",
`sPLA2_IIATGN__>_WT` =  "#32CD32",
            `WT__>_sPLA2_IIATGN` = "#FF8C00" , `WT__>_WT` =
"#1E90FF" ),
  Timepoint = c(`D0_(Original)` = "#edf8fb", `D15_(Pre_arthritis)` =
"#b2e2e2",
                `D21_(Pre_arthritis)` = "#66c2a4",`D29_(Arthritis)` =
"#238b45")
  #Colony = c(B6.sPLA2_IIATGN = "#fa9fb5",`C57BL/6J` = "#c51b8a"),
  #X._Mouse = X._Mouse_tab
)
b.pca <- prcomp(betaDistMatrix, scale. = F)
autoplot(b.pca, data = anno_row, colour = 'Group', shape =
'Timepoint',frame = FALSE,size = 7, frame.type = 'norm', label =
FALSE, label.size = 4, label.face = "bold" ) +
  scale_colour_manual(values = my_colour$Group)+
  theme(legend.position="bottom",strip.text = element_text(size=12,
face = "bold"), legend.text = element_text(size=14, face = "bold"))
```

Differential analysis aldex
```
cond_transfD1=filter(coldata, str_detect(Group, "Donor") & Timepoint
== "D0_(Original)" | Group %in% c("TgIIA__>_TgIIA",
"TgIIA__>_WT","WT__>_TgIIA", "WT__>_WT") & Timepoint %in%
c("D29_(Arthritis)","D21_(Pre_arthritis)") )
cond_transfD1$inflamation_response = if_else(cond_transfD1$Group %in%
c("Donor_TgIIA","TgIIA__>_TgIIA"), "Inflamed", "Non-inflamed")
abun_transfD1 = dplyr::select(abun_filt, cond_transfD1$sample_name)
stopifnot(all(colnames(abun_transfD1) == cond_transfD1$sample_name))
cond2 = cond_transfD1$inflamation_response

aldex_dds = aldex.clr(abun_transfD1, cond2, mc.samples = 128, denom =
"all", verbose=TRUE)
aldex_dds.tt <- aldex.ttest(aldex_dds, verbose=TRUE)
aldex_dds.effect <- aldex.effect(aldex_dds, verbose=TRUE)
aldex_dds.all <- data.frame(aldex_dds.tt,aldex_dds.effect)
res = filter(aldex_dds.tt, we.eBH < 1) %>% arrange(we.eBH) %>%
rownames_to_column()
```

visualization of different ASVs

```

resfil = left_join(filter(rownames_to_column(aldex_dds.tt, "ASVs"),
we.eBH < 0.05), blast, by = "ASVs")
resfil = left_join(resfil, tax, by = "geneID")
resfil = left_join(resfil, names_dmp, by = "taxid")
resfil2 = resfil[base::order(resfil$we.eBH),]
resfil2$label =
paste(paste(resfil2$ASVs,"pval:",format(resfil2$we.eBH,
scientific=TRUE, digits=2), sep = " "), resfil2$name_tax,
paste(format(resfil2$percent,digits = 2), "% identity",sep = " ") ,
sep ="\n")
d_long = rownames_to_column(abun_transfD1)  %>% pivot_longer(-rowname)
%>% filter(rowname %in% resfil2$ASVs)
colnames(d_long) = c("ASVs","sample_name","value")
d_long = left_join(d_long, cond_transfD1, by = "sample_name")
d_long = left_join(d_long, resfil2, by = "ASVs")
d_long$label = factor(d_long$label, levels = c(resfil2$label))
test = d_long %>% group_by(inflamation_response, ASVs) %>%
mutate(somme = sum(value))
test$label2 = str_replace(test$label, "Muribaculum intestinale",
"Uncultured Muribaculum sp." )
test$label2 = str_remove(test$label2, "\n 91 % identity" )
test$label2 = str_replace_all(test$label2, "\n91 % identity", "\n100 %
identity" )
test$Group = str_replace(test$Group, "__>_", " flora->strain " )
test$Group = str_replace(test$Group, "_", " " )
test$Group = str_replace_all(test$Group, "TgIIA", "sPLA2-IIATGN" )
```

```
test$Timepoint = str_replace(test$Timepoint, "_\\(" , "\n" )
test$Timepoint = str_replace(test$Timepoint, "\\)" , "" )

ggplot(data= test ) +
    aes(x=inflamation_response, y=value, ) +xlab("indidivual") +
ylab("Distribution of ASVs")+ #fill = label
    theme(legend.position = "none",strip.text = element_text(size=12,
face ="bold"),axis.text.x = element_text(size = 10, angle = 45,
hjust=1))+
    #geom_bar(stat="identity") +
    geom_boxplot()+

#facet_grid(~exp~group ,labeller=label_parsed,scales="free",space="fre
e")+

#facet_nested(label2~inflamation_response+Group+Timepoint ,scales="fre
e", drop = FALSE)
  facet_wrap(~label ,scales="free")
```
