# Supplementary material for: The interaction of secreted phospholipase A2-IIA with the microbiota alters its lipidome and promotes inflammation
Source: JCI Insight. 2022 Jan 25;7(2):e152638. doi: 10.1172/jci.insight.152638 (PMC8855825; doi:10.1172/jci.insight.152638)
Supplement: Supplemental table 6 [file jciinsight-7-152638-s160.pdf]

| | "dada2_input" | "filtered" | "dada_f" | "dada_r" | "merged" | "nonchim" | "final_perc_reads_retained" |
|---|---|---|---|---|---|---|---|
| "26398-Donor_S1" | 78898 | 62338 | 61766 | 62032 | 57008 | 29322 | 37.2 |
| "26399-Donor_S2" | 75488 | 59178 | 58371 | 58763 | 51983 | 26605 | 35.2 |
| "26491-Donor_S8" | 38661 | 31196 | 30766 | 30981 | 27330 | 12079 | 31.2 |
| "26492-Donor_S9" | 74286 | 59843 | 59041 | 59382 | 52892 | 24456 | 32.9 |
| "26493-Donor_S10" | 85836 | 67271 | 66374 | 66715 | 58822 | 25277 | 29.4 |
| "26494-Donor_S11" | 75534 | 60422 | 59595 | 59996 | 53883 | 25122 | 33.3 |
| "26495-Donor_S12" | 100817 | 80205 | 78936 | 79635 | 70272 | 32333 | 32.1 |
| "26496-Donor_S3" | 80392 | 63826 | 62853 | 63349 | 54867 | 25360 | 31.5 |
| "26573-Donor_S13" | 83004 | 66343 | 65428 | 65898 | 59016 | 25812 | 31.1 |
| "26574-Donor_S14" | 75096 | 60573 | 59913 | 60260 | 54554 | 24631 | 32.8 |
| "26575-Donor_S4" | 83348 | 65752 | 64719 | 65280 | 57355 | 28605 | 34.3 |
| "26576-Donor_S5" | 60186 | 46239 | 45811 | 45933 | 42215 | 22565 | 37.5 |
| "26577-Donor_S6" | 69557 | 54412 | 53979 | 54164 | 50339 | 27645 | 39.7 |
| "26701-Donor_S7" | 79426 | 62300 | 61503 | 61830 | 54665 | 22990 | 28.9 |
| "26706-D0_S30" | 63610 | 51324 | 50465 | 50866 | 42727 | 20872 | 32.8 |
| "26706-D21_S51" | 85381 | 69824 | 68971 | 69197 | 61516 | 29204 | 34.2 |
| "26706-D29_S71" | 54663 | 44854 | 43909 | 44456 | 38242 | 17824 | 32.6 |
| "26707-D0_S20" | 67064 | 50866 | 50187 | 50554 | 43697 | 20141 | 30 |
| "26707-D21_S41" | 82789 | 65482 | 64411 | 64866 | 56245 | 24519 | 29.6 |
| "26707-D29_S61" | 83125 | 66127 | 64984 | 65617 | 58876 | 34553 | 41.6 |
| "26761-D0_S21" | 73134 | 55444 | 54641 | 54978 | 47255 | 21908 | 30 |
| "26761-D21_S42" | 59851 | 46510 | 46056 | 46249 | 42257 | 20820 | 34.8 |
| "26761-D29_S62" | 73550 | 57544 | 56735 | 57241 | 51437 | 24634 | 33.5 |
| "26762-D0_S22" | 67287 | 49075 | 48585 | 48846 | 44283 | 19542 | 29 |
| "26762-D21_S43" | 78478 | 60289 | 59632 | 59966 | 55065 | 29768 | 37.9 |
| "26762-D29_S63" | 95936 | 75898 | 74932 | 75374 | 68270 | 33625 | 35 |

| | | | | | | | |
|---|---|---|---|---|---|---|---|
| "26763-D0_S31" | 64263 | 52591 | 51593 | 52118 | 43736 | 19416 | 30.2 |
| "26763-D21_S52" | 89976 | 73099 | 71867 | 72442 | 61683 | 28549 | 31.7 |
| "26763-D29_S72" | 62721 | 50054 | 49302 | 49544 | 42836 | 20736 | 33.1 |
| "26783-D0_S15" | 84714 | 64354 | 62994 | 63718 | 53884 | 23109 | 27.3 |
| "26783-D21_S37" | 68531 | 54979 | 54462 | 54699 | 49564 | 24999 | 36.5 |
| "26783-D29_S56" | 64932 | 51621 | 50857 | 51199 | 44597 | 20848 | 32.1 |
| "26784-D0_S16" | 71656 | 54591 | 53795 | 54152 | 48214 | 21073 | 29.4 |
| "26784-D21_S38" | 76553 | 58949 | 58512 | 58667 | 53804 | 26629 | 34.8 |
| "26784-D29_S57" | 79266 | 61752 | 61078 | 61390 | 55239 | 29251 | 36.9 |
| "26785-D0_S25" | 62159 | 46088 | 45419 | 45766 | 40541 | 18332 | 29.5 |
| "26785-D21_S46" | 74910 | 58642 | 57937 | 58231 | 51903 | 24344 | 32.5 |
| "26785-D29_S66" | 82678 | 65936 | 65013 | 65477 | 58046 | 27042 | 32.7 |
| "26786-D0_S26" | 69322 | 56063 | 55123 | 55442 | 48082 | 20573 | 29.7 |
| "26786-D21_S47" | 78818 | 63563 | 62843 | 63195 | 56809 | 26456 | 33.6 |
| "26786-D29_S67" | 97887 | 77769 | 76914 | 77235 | 70055 | 33015 | 33.7 |
| "26787-D0_S27" | 63905 | 39073 | 38406 | 38801 | 33058 | 14599 | 22.8 |
| "26787-D21_S48" | 66155 | 54428 | 53551 | 53986 | 47122 | 21819 | 33 |
| "26787-D29_S68" | 86919 | 70644 | 69503 | 69986 | 60756 | 28677 | 33 |
| "26789-D0_S23" | 76994 | 62308 | 61640 | 61978 | 55948 | 22839 | 29.7 |
| "26789-D21_S44" | 73370 | 56774 | 56316 | 56517 | 52110 | 26700 | 36.4 |
| "26789-D29_S64" | 72457 | 55891 | 55137 | 55488 | 49552 | 23738 | 32.8 |
| "26790-D0_S28" | 84980 | 68996 | 68024 | 68525 | 59910 | 25597 | 30.1 |
| "26790-D21_S49" | 81463 | 65185 | 64503 | 64799 | 59086 | 30000 | 36.8 |
| "26790-D29_S69" | 81022 | 65350 | 64593 | 64875 | 58299 | 30356 | 37.5 |
| "26791-92-D21_S39" | 91779 | 72839 | 71936 | 72293 | 63416 | 29951 | 32.6 |
| "26791-D0_S17" | 79590 | 60182 | 59192 | 59750 | 50790 | 23573 | 29.6 |

| | | | | | | | |
|---|---|---|---|---|---|---|---|
| "26791-D15_S35" | 80764 | 62124 | 61624 | 61786 | 57512 | 32551 | 40.3 |
| "26791-D29_S58" | 75333 | 59037 | 58462 | 58626 | 53594 | 28710 | 38.1 |
| "26792-D0_S18" | 72999 | 54954 | 53914 | 54409 | 45103 | 20811 | 28.5 |
| "26792-D15_S36" | 71213 | 57174 | 56722 | 56796 | 52928 | 29636 | 41.6 |
| "26792-D29_S59" | 76107 | 59718 | 58814 | 59249 | 53006 | 29247 | 38.4 |
| "26797-D0_S32" | 80294 | 66166 | 65108 | 65661 | 57285 | 24289 | 30.3 |
| "26797-D21_S53" | 92563 | 75845 | 74746 | 75169 | 68273 | 34994 | 37.8 |
| "26797-D29_S73" | 80328 | 64857 | 64004 | 64315 | 57401 | 26469 | 33 |
| "26798-D0_S33" | 85300 | 67852 | 66817 | 67339 | 60175 | 25834 | 30.3 |
| "26798-D21_S54" | 94236 | 74386 | 73344 | 73649 | 66426 | 35705 | 37.9 |
| "26798-D29_S74" | 74512 | 60396 | 59566 | 59994 | 53378 | 22354 | 30 |
| "26833-D0_S29" | 96891 | 78902 | 77884 | 78257 | 70069 | 29855 | 30.8 |
| "26833-D21_S50" | 79537 | 64910 | 64093 | 64418 | 57889 | 26412 | 33.2 |
| "26833-D29_S70" | 56521 | 44648 | 43860 | 44251 | 37796 | 17912 | 31.7 |
| "26834-D0_S19" | 92437 | 69448 | 68406 | 68842 | 60302 | 25281 | 27.3 |
| "26834-D21_S40" | 56732 | 44475 | 44006 | 44189 | 39225 | 21869 | 38.5 |
| "26834-D29_S60" | 73444 | 57299 | 56619 | 56933 | 50785 | 26264 | 35.8 |
| "26877-D0_S34" | 72577 | 59569 | 59018 | 59215 | 54719 | 24083 | 33.2 |
| "26877-D21_S55" | 61457 | 48865 | 48089 | 48463 | 41353 | 22019 | 35.8 |
| "26877-D29_S75" | 80101 | 64646 | 63743 | 64201 | 55735 | 24240 | 30.3 |
| "26878-D0_S24" | 76275 | 62570 | 61670 | 62085 | 54067 | 23395 | 30.7 |
| "26878-D21_S45" | 82269 | 65640 | 64414 | 64873 | 56283 | 27205 | 33.1 |
| "26878-D29_S65" | 110475 | 86979 | 85831 | 86410 | 78868 | 40713 | 36.9 |
